# Comprehensive characterization of cardiac contraction for improved post-infarction risk assessment

**DOI:** 10.1038/s41598-024-59114-3

**Published:** 2024-04-18

**Authors:** Jorge Corral Acero, Pablo Lamata, Ingo Eitel, Ernesto Zacur, Ruben Evertz, Torben Lange, Sören J. Backhaus, Thomas Stiermaier, Holger Thiele, Alfonso Bueno-Orovio, Andreas Schuster, Vicente Grau

**Affiliations:** 1 Department of Engineering Science, Institute of Biomedical Engineering, University of Oxford, Oxford, UK; 2 Department of Digital Twins for Healthcare, School of Biomedical Engineering and Imaging Sciences, King’s College London, 4th Floor North Wing, St Thomas’ Hospital, London, SE1 7EH UK; 3 Medical Clinic II, Cardiology, Angiology and Intensive Care Medicine, University Heart Centre Lübeck, Lübeck, Germany; 4 University Hospital Schleswig-Holstein, Lübeck, Germany; 5 German Centre for Cardiovascular Research (DZHK), Partner Site Hamburg/Kiel/Lübeck, Lübeck, Germany; 6 Department of Cardiology and Pneumology, University Medical Centre Göttingen, Georg-August University, Göttingen, Germany; 7 German Centre for Cardiovascular Research (DZHK), Partner Site Lower Saxony, Göttingen, Germany; 8 Department of Cardiology, Campus Kerckhoff of the Justus-Liebig-University Giessen, Kerckhoff-Clinic, Bad Nauheim, Germany; 9 German Center for Cardiovascular Research (DZHK), Partner Site Rhine-Main, Bad Nauheim, Germany; 10 Department of Internal Medicine/Cardiology and Leipzig Heart Science, Heart Centre Leipzig at University of Leipzig, Leipzig, Germany; 11 Department of Computer Science, University of Oxford, Oxford, UK

**Keywords:** Computational biology and bioinformatics, Biomarkers, Cardiology, Risk factors, Mathematics and computing

## Abstract

This study aims at identifying risk-related patterns of left ventricular contraction dynamics via novel volume transient characterization. A multicenter cohort of AMI survivors (n = 1021) who underwent Cardiac Magnetic Resonance (CMR) after infarction was considered for the study. The clinical endpoint was the 12-month rate of major adverse cardiac events (MACE, n = 73), consisting of all-cause death, reinfarction, and new congestive heart failure. Cardiac function was characterized from CMR in 3 potential directions: by (1) volume temporal transients (i.e. contraction dynamics); (2) feature tracking strain analysis (i.e. bulk tissue peak contraction); and (3) 3D shape analysis (i.e. 3D contraction morphology). A fully automated pipeline was developed to extract conventional and novel artificial-intelligence-derived metrics of cardiac contraction, and their relationship with MACE was investigated. Any of the 3 proposed directions demonstrated its additional prognostic value on top of established CMR indexes, myocardial injury markers, basic characteristics, and cardiovascular risk factors (*P* < 0.001). The combination of these 3 directions of enhancement towards a final CMR risk model improved MACE prediction by 13% compared to clinical baseline (0.774 (0.771—0.777) vs. 0.683 (0.681—0.685) cross-validated AUC, *P* < 0.001). The study evidences the contribution of the novel contraction characterization, enabled by a fully automated pipeline, to post-infarction assessment.

## Introduction

Cardiovascular diseases are the worldwide leading cause of death, with a toll further climbing each decade^1^. Acute myocardial infarction (AMI) plays a major role among them, owing to its prevalence, counted in millions, yearly, and its high case-fatality^2^. Despite significant advances in medical treatment towards personalized and preventive medicine^2,3^, the mortality of AMI survivors within 6 months after infarct sits at 12%^4^. Early and improved risk assessment is crucial to reduce this burden.

Left ventricular (LV) remodeling is central to AMI early prognosis prediction^5^. Consequently, LV macro-function, quantified as LV ejection fraction (LVEF), is the preferred image biomarker for therapeutic decision‐making and clinical risk stratification, according to AMI guidelines^4,6^. Nonetheless, LVEF fails to capture contraction dynamics as well as functional and anatomical regional abnormalities^7,8^. Moreover, LVEF is mainly preserved or only moderately reduced in most AMI survivors and therefore recurrent adverse events often occur in patients at a theoretical low risk, which further evidences the need of stratification improvements^9,10^.

Cardiac Magnetic Resonance (CMR) imaging has proven uniquely suitable to assess morphological and functional myocardial alterations and provides prognostic information over and above LVEF^11–13^. Despite its cost and acquisition length limitations, the level of precision of CMR has established it as gold standard for quantitative assessment of the heart. Furthermore, CMR uses no ionizing radiation unlike computed tomography imaging^14^. LV micro-myocardial injury, assessed by late gadolinium enhancement (LGE) CMR and usually quantified as infarct size (IS) and microvascular obstruction (MVO), has emerged as a robust outcome measure in AMI risk assessment^13,15^. Estimation of local LV remodeling using CMR has been proposed to improve prognostic information^16,17^. Likewise, shape analysis of the LV to identify 3D patterns related to risk has demonstrated its additional contribution to risk assessment^18^. CMR myocardial feature tracking (CMR-FT) has been successfully applied for quantification of LV deformation indexes and mechanical uniformity alterations^7,19^, associated with hemodynamic alterations, adverse LV remodeling, and clinical outcomes^9,20^. In particular, the prognostic value of global longitudinal strain (GLS) by CRM-FT has proven superior and incremental to LVEF and other CMR markers of infarct severity^21^. Recent advances in deep learning enable the extraction of CMR-derived volume temporal transients, which have proven to be a robust and useful biomarker to characterize cardiac function^22^.

**Figure 1:**
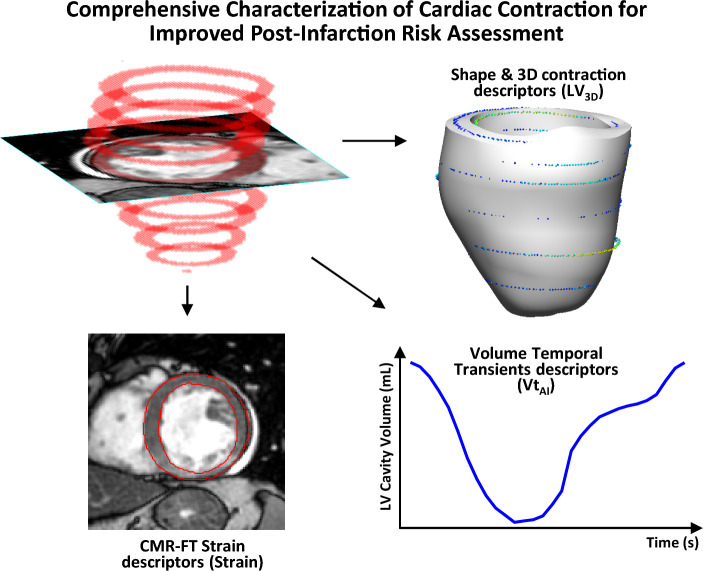
Comprehensive Characterization of Cardiac Contraction for Improved Post-Infarction Risk Assessment. The 3 proposed directions of enhancement of cardiac contraction characterization from CMR, enabled by a fully automated pipeline, for improved post-infarction management beyond left ventricular ejection fraction (LVEF), the established marker.

Building on these advances, and further automating and enhancing the descriptors of contraction, this work focuses on the ability to detect novel signatures that predict post-infarction risks from the temporal changes observed at chamber level (i.e. LV volume transients). The work additionally explores the complementary value of tissue level dynamics (i.e. strains) and, also, the static 3D detail that we have recently demonstrated to bring prognostic value in our previous work^18^, an analysis that is limited to the end-systolic (ES) and end-diastolic (ED) instances. Here we expand on the work in^17^ and complete it by applying related ideas to the characterization of CMR-FT strains and the novel use of LV volume transients for post-infarction risk assessment. We assess the prognostic contribution of this novel cardiac contraction characterization using all three image-based components (strains, transients and 3D LV patterns) on a large multicenter study, including ST‐segment–elevation myocardial infarction (STEMI) and non‐STEMI (NSTEMI) patients (see Fig. 1). The study demonstrates the potential of the herein proposed fully automated analysis for AMI risk management and identifies novel risk-related contraction patterns, expanding the understanding of post-infarction remodeling.

## Methods

### Study population

A total of 1235 AMI survivors from the AIDA-STEMI and TATORT-NSTEMI multicenter randomized trials were considered for the study^23–25^. The trials were registered with ClinicalTrials.gov (Registration ID and date: AIDA-STEMI: NCT00712101, 03/07/2008; TATORT-NSTEMI: NCT01612312, 01/06/2012) and their experimental protocols were approved by the lead ethical committee at the University of Leipzig and by all the local ethics and licensing committees of the participating sites (Zentralklinik Bad Berka, Unfallkrankenhaus Berlin, Klinikum Frankfurt/Oder, University of Saarland, Institut für Herzinfarktforschung, University of Tübingen, Herz- und Gefäß-KLinik Bad Neustadt, Herz und Diabeteszentrum Bad Oeynhausen, Klinikum Links der Weser Bremen, Klinikum Coburg, Carl-von-Basedow-Klinikum Merseburg, Klinikum Pirna, Krankenhaus der Barmherzigen Brüder, Jochen Wöhrle and Klinikum der Stadt Villingen-Schwenningen). This size of the study is justified in Supplemental Material VIII, based on^26^. Reperfusion therapy with primary percutaneous coronary intervention and postinfarction medical treatment were supplied according to state-of-the-art guideline recommendations^4^. The study was conducted according to the Declaration of Helsinki and written informed consent was obtained from all subjects and/or their legal guardian for study participation. The data that supports the findings is available upon reasonable request.

### CMR imaging protocol

The patients underwent CMR imaging on 1.5T (Siemens models: Aera, Avanto, Espree, Sonata, SonataVision and Symphony; Philips models: Achieva and Intera; GE models: Signa excite) or 3T (Siemens Verio) clinical scanners within 10 days after infarct following a standardized protocol^6,23,24^, that includes ECG-gated balanced steady-state free precession sequences (TR = 3.573 ms; TE median of 1.786 (1.649—1.786) ms; Flip angle = 60°) and T1-weighted LGE images. All sequences were acquired in horizontal and vertical long-axis views as well as continuous short-axis (SAx) stacks capturing the whole LV (Pixel size: 1.25 (1.25—1.48) mm; Spacing between SAx: 8.00 (8.00–8.00) mm). Ventricular volumes and infarct characteristics were determined in sequential SAx by blinded clinicians^6,24^, via dedicated software cmr42^27^. Standard thresholding techniques were applied to assess IS and MVO, as explained in^6,24^.

### Study endpoints

The predefined clinical endpoint of the study was the 12-month rate of major adverse cardiac events (MACE), consisting of new congestive heart failure, reinfarction and all-cause death, as detailed in^23–25^. In case of multiple MACE only one contribution per patient to the composite of endpoints was considered (death > reinfarction > congestive heart failure). The events were adjudicated by a blinded clinical committee based on the data collected in the study sites.

### CMR-FT strain analysis (strain)

CMR-FT was conducted at the University Medical Centre Göttingen core laboratory^28^, using certified software 2D Cardiac Performance Analysis MR^29^. Final values were based on the average of 3 independent analyses^30^. Circumferential and radial strains were determined at basal, midventricular, and apical locations, as described in^9,20^. Longitudinal strains were derived at both 2- and 4-chamber long-axis views. Global longitudinal, circumferential, and radial strains (GLS, GCS and GRS, respectively) as well as circumferential and radial uniformity ratio estimates (CURE and RURE), were reported^31^. CURE and RURE range between 0, complete nonuniform contraction, and 1, perfect uniformity, that is, equal strain across the myocardium at any given time point. Calculation details can be found in^9^.

### LV fully automated volume transients analysis

**Figure 2:**
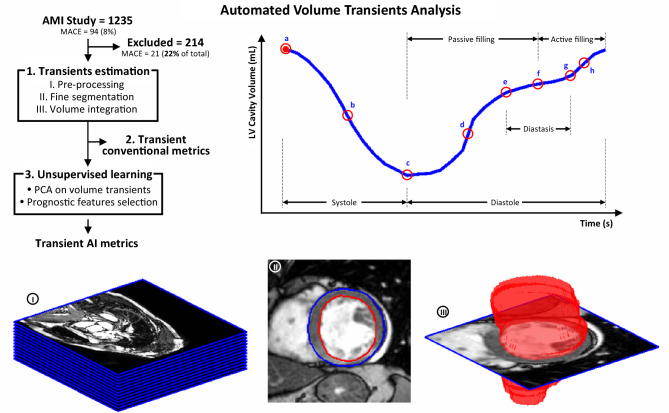
Automated volume transient analysis. LV fully automated volume transient analysis pipeline (top-left), along with patient S692 sample case, consisting of the following steps: (1.I.) the SAx stack (image I) is pre-processed normalizing for intensity, resolution and orientation and cropped to the region of interest, following the prior detection of the heart; (1.II.) the LV myocardium is fine segmented in the SAx stack (image II); (1.III) a volumetric segmentation is synthesized (image III) and the LV volume transient derived; (2) the conventional metrics of the volume transient are estimated (top-right); and (3) PCA analysis is applied on the transients to facilitate the supervise learning of MACE occurrence related features (AI-derived transient metrics). The red circle landmarks on the representative volume transient (top-right) correspond to: a, end-diastole; b, systolic peak velocity; c, end-systole; d, passive filling peak velocity; e, beginning of diastasis; f, mid-diastasis; g, end-diastasis; and h, active filling peak velocity. This figure is available in video format (see Supplementary Video 1).

The process, illustrated in Fig. 2, is fully automated and consist of the following steps: (1) LV myocardium segmentation from SAx images and LV volume temporal transient reconstruction from the cavity volume integration along the cardiac cycle; (2) Computation of conventional metrics from LV volume transient; (3) Definition of Artificial Intelligence (AI) based metrics of LV volume transients, by unsupervised construction of statistical models and the subsequent selection of the features with best prognostic value.

#### LV segmentation and volume transients estimation

A 2-step deep learning approach was applied to segment the LV cavity, based on a UNet architecture with enhanced pre-processing^32,33^, that reached the best performance in the 2019 LV Full Quantification Challenge^33,34^. Implementation details are available in Supplemental Material I. Resulting segmentations were arranged in binary volumetric images, and the LV cavity volume was derived for each frame via trapezoidal integration.

#### Volume transient conventional metrics (Vt)

The duration, average and peak velocity of the cardiac phases shown in Fig. 2 were derived^22^. The transients were resampled using splines to increase resolution and better capture the slope-sensitive metrics. Diastasis was estimated as the mid-diastolic region with a velocity inferior to 80% stroke volume (SV) per second (empirical threshold). The time to diastasis, measured from end-systole and mid-diastasis, was set as the boundary between the passive and active filling contributions. Passive and active filling metrics were not reported in those cases where diastasis was not reliably found (diastasis = 0s , n = 212). The average RR-interval and its variability, measured as highest minus lowest values, as well as the frame trigger times, were directly retrieved from the patient scans metadata.

#### Volume transient AI metrics (Vt_AI_)

**Figure 3:**
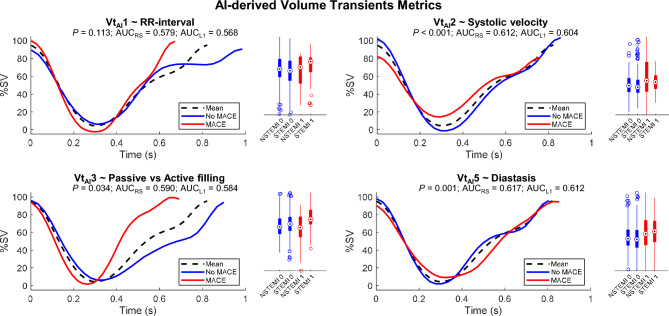
AI-derived volume transient metrics. AI-derived volume transient features most relevant to MACE occurrence prediction, resulting from unsupervised learning (Vt_AI_1, Vt_AI_2, Vt_AI_3 and Vt_AI_5). The MACE (red, class 1) and No-MACE (blue, class 0) traces shown correspond to the 10^th^ and 90^th^ percentiles. This allows to visualize the pattern of change encoded by each of the unsupervised variables (RR-interval, passive vs active filling, etc.), as well as to describe how a representative MACE and No-MACE normalized volume transient would theoretically look like according to each of these four unsupervised variables. The *P*-value, re-substitution and leave-one-out AUCs are presented along each mode as MACE and No-MACE distributions, further stratified into infarct aetiology (STEMI and NSTEMI). This figure is available in video format, where the evolution from the MACE to the No MACE extremes is shown to better appreciate the pattern of change encoded in each of the VT_AI_ contraction modes (see Supplementary Video 2).

Principal Component Analysis (PCA) is an unsupervised machine learning technique that inspects the data to find the directions of change that maximize the variance observed in the population. The main advantages of the PCA strategy are: (I) the ability to extract interpretations from the linear space of features; (II) the unsupervised definition of features, that are unbiased to outcome prediction; (III) the ability to minimize the impact of noise and the reduced risk of overfitting by working with the principal modes of variation. Independently from the above calculations on raw volume transients, PCA was applied to the volume transients (Formulation details and a more detailed explanation is available in Supplemental Material III), minmax normalized to standardize in ventricle size and SV (see implications in Supplemental Material II). The resulting directions are the so called ‘PCA modes’, and represent patterns of change with respect to the mean volume transients (e.g. RR-interval length, passive vs. active contribution, etc.—See Fig. 3, panel headings representing mode interpretation). Thus, the volume transient of each subject can be represented by the average transient of the population plus a weighted sum of these modes of variation. The weights used are, therefore, continuous variables which have a unique value for each patient and become potential biomarkers to predict MACE. Among them, those with highest prognostic value were identified by a stepwise multivariable Fisher linear discriminant analysis (LDA)^35^, as detailed below, on the modes that reconstruct 95% of the total variance of the population.

### LV fully automated 3D shape analysis (LV_3D_)

The analysis is explained in^18^. In brief, an automated pipeline was developed to automatically obtain the personalized 3D LV mesh at ES and ED; and a similar AI methodology to the explained above is applied to identify the MACE-related ES shape and 3D contraction patterns. The contraction was approximated as ED to ES displacements. Table 1 summarizes the three ES shape and three contraction descriptors found to be prognostic, along with their interpretation^18^. These 3D LV patterns are illustrated in Fig. SIV.1.

**Table 1:** - Baseline CMR, Baseline ALL and 3D Left-ventricular variables.

	Nos
CMR—Baseline CMR variables	
End-systolic volume (mL), End-diastolic volume (mL), Left ventricular ejection fraction (%), Infarct size (mL), Infarct size (%LV mass), Microvascular obstruction (mL)	6
ALL—Baseline ALL variables	
Age (y), Body mass index (Kg/m2), Body surface area (m2), Current smoking, Diabetes, Height (cm), Hyperlipoproteinemia, Hypertension, Killip class on admission, Number of disease vessels, Sex, TIMI flow grade post-PCI, Weight (kg) and the 6 CMR variables indicated above	19
LV_3D_—3D Left-ventricular End-systolic shape and Contraction variables	
Mode ES1 (~ Global Impairment, ESV), Mode ES5 (~ Anterior Impairment), Mode ES6 (~ Impaired Thickening), Mode C3 (~ Global Impairment, LVEF), Mode C5 (~ Anterior Impairment), Mode C16 (~ Basal Impairment)	6

List and number of variables included in the baseline CMR (CMR), baseline ALL (ALL) and LV 3D patterns (LV_3D_) groups for prognostic assessment experiments. Interpretation of each of the LV_3D_ variables is indicated in parenthesis. See^18^ for more details.

### Prognostic value assessment

The additional prognostic contribution of each of the 3 proposed directions of enhancement, CMR-FT strains (strains), volume temporal transients (conventional and AI-derived—Vt and Vt_AI_) and LV 3D patterns (LV_3D_) markers, was assessed considering only CMR biomarkers (CMR) and all the clinical variables (ALL) of the study. Table 1 lists the ‘CMR’ (e.g. MVO, IS, etc.) and ‘ALL’ (e.g. age, weight, etc.) baseline variables included in the analysis as predictor candidates. An additional analysis evaluated the prognostic value of the three proposed groups of contraction metrics in combination.

Comparisons were based on the prediction performance resulting from both a multivariable LDA (binary classification–MACE occurrence at 12 months) and a multivariable Cox analysis (time to MACE occurrence). A backward stepwise strategy (unbiased to univariable associations^36^) was followed in both analyses to find inter-variable synergies and address collinearity: all variables of interest (predictor candidates) were initially included, and the less significant in the model iteratively removed, until a 0.05 *p*‐value threshold was met by all of them. Significance in the LDA was approximated as significance in the generalized linear binomial sigmoid regression model.

The performance of the resulting configurations was assessed via area under the receiver-operator characteristic curve (AUC), for binary classifications, and concordance index (C-index)^37^, for time-dependent curves, to account for both specificity and sensitivity, given the MACE class imbalance. Performance in both resubstitution (apparent performance—learning and testing with the entire cohort) and cross-validation (optimism corrected performance—10 cross-fold-validation repeated for a hundred random data splits) is reported to ensure generality of findings.

### Statistical analysis

The variables of the study are described according to MACE occurrence (Table 2). Continuous variables were not normally distributed in a Shapiro–Wilk test, except for the Vt_AI_ modes of variation (Gaussian distributed by definition), and are therefore presented as median and interquartile range (IQR). Distributions (variable stratifications, cross-validated AUC model results, etc.) were compared by the non-parametric Wilcoxon rank sum test. The resulting *p*-value of the MACE vs No MACE comparisons, as well as the univariate-LDA cross-validated performance, was reported for each variable. Univariate Cox regression analyses were likewise completed. Hazard ratios, 95% confidence intervals and the predictor significance are presented in the results. Correlation between variables was assessed via Pearson (r_p_) and Spearman (r_s_) coefficients. Patients with missing data in any of the model variables are excluded for the development of this particular model. All analyses were implemented in Matlab^38^, except for neural network training and inference; that were implemented in Keras with Tensorflow as backend^39^. The experiments and analyses were run on a standard laptop (RAM: 32Gb; GPU: 16Gb). The study follows the guidelines of transparent reporting of multivariable prediction model for individual prognosis or diagnosis (TRIPOD) as well as the Strengthening the Reporting of Observational Studies in Epidemiology (STROBE) Statement for case–control studies^36,40^. The relevant checklists are included in Supplemental Material VIII.

**Table 2:** CMR-FT strain, conventional and AI-derived automated volume transient metrics.

Variable	ALL patients	MACE (n = 73)	No MACE (n = 948)	AUC_k_	*P*val	HR	HR *Pval*
Strain–Strain markers							
Global longitudinal strain (%)	− 16.5 (− 20.2 to− 12.5)	− 11.5 (− 16.8–-8.4)	− 16.7 (− 20.4 to − 13.0)	0.710	< 0.001	2.13 (1.68–2.69)	< 0.001
Global circumferential strain (%)	− 24.2 (− 28.9 to − 19.2)	− 18.6 (− 24.2–-14.6)	− 24.4 (− 29.1 to − 19.7)	0.674	< 0.001	1.72 (1.39–2.12)	< 0.001
Global radial strain (%)	20.4 (15.7–25.9)	16.4 (12.6–22.6)	20.5 (16.0–26.0)	0.626	< 0.001	0.64 (0.50–0.83)	< 0.001
CURE	0.78 (0.71–0.84)	0.71 (00.7–0.80)	0.79 (0.72–0.84)	0.637	< 0.001	0.62 (0.50–0.76)	< 0.001
RURE	0.75 (0.67–0.82)	0.69 (00.6–0.78)	0.76 (0.67–0.83)	0.629	< 0.001	0.66 (0.54–0.82)	< 0.001
Vt—Volume transient markers							
RR-interval (ms)	845 (741–952)	780 (674–848)	845 (750–952)	0.644	< 0.001	0.64 (0.51–0.81)	< 0.001
RR-interval variability (%RR)	19.2 (13.1–26.3)	24.6 (14.2–36.7)	18.9 (13.0–25.6)	0.606	< 0.001	1.28 (1.13–1.46)	< 0.001
Systolic phase							
Systolic time (ms)	297 (267–328)	284 (265–321)	298 (268–328)	0.562	0.106	0.70 (0.51–0.98)	0.038
Systolic velocity avg (mL/s)	251 (209–301)	227 (184–271)	252 (212–303)	0.592	0.238	0.61 (0.34–1.11)	0.105
Systolic velocity avg (%SV/s)	337 (305–375)	352 (312–377)	336 (305–373)	0.554	0.314	1.08 (0.93–1.25)	0.315
Systolic velocity peak (mL/s)	570 (451–704)	514 (396–650)	573 (456–711)	0.573	0.030	0.77 (0.60–0.97)	0.029
Diastolic phase							
Diastolic time (ms)	513 (431–602)	462 (394–523)	518 (435–606)	0.611	0.028	0.76 (0.60–0.97)	0.025
Diastasis (ms)	38 (0–118)	24 (0–83)	44 (0–119)	0.569	0.034	0.74 (0.56–0.98)	0.037
Diastasis (%)	10.8 (0.0–21.6)	5.4 (0.0–16.2)	10.8 (0.0–21.6)	0.558	0.030	0.74 (0.56–0.97)	0.032
Time to diastasis (ms)	354 (307–402)	327 (267–366)	356 (309–405)	0.605	0.017	0.70 (0.53–0.93)	0.013
Time to diastasis (%)	64.9 (62.2–70.3)	64.9 (56.8–70.9)	64.9 (62.2–70.3)	0.512	0.192	0.82 (0.62–1.09)	0.177
Passive filling (mL)	55.6 (43.0–67.2)	48.1 (37.6–60.1)	56.0 (43.5–67.9)	0.630	0.002	0.64 (0.48–0.85)	0.002
Passive filling (%SV)	66.5 (58.2–73.5)	67.3 (57.8–78.9)	66.4 (58.2–73.0)	< 0.5	0.767	1.05 (0.77–1.42)	0.776
Active filling (mL)	26.2 (19.9–33.6)	20.6 (12.7–28.5)	26.7 (20.2–33.9)	0.647	< 0.001	0.71 (0.59–0.87)	< 0.001
Active filling (%SV)	31.5 (23.8–39.8)	26.6 (17.8–37.7)	31.7 (24.1–39.9)	0.559	0.110	0.81 (0.63–1.04)	0.094
Diastolic velocity avg (mL/s)	143 (121–170)	141 (109–168)	144 (122–170)	0.535	0.458	0.20 (0.04–0.97)	0.046
Diastolic velocity avg (%SV/s)	195 (166–232)	216 (191–254)	193 (165–230)	< 0.5	0.982	1.00 (0.79–1.26)	0.982
Passive filling velocity avg (mL/s)	155 (122–187)	151 (118–180)	155 (122–188)	0.516	0.291	0.87 (0.69–1.11)	0.274
Passive filling velocity avg (%SV/s)	185 (153–215)	205 (170–239)	184 (153–213)	0.592	0.015	1.35 (1.07–1.71)	0.013
Passive filling velocity peak (mL/s)	427 (307–573)	384 (271–502)	429 (310–575)	0.553	0.143	0.80 (0.59–1.08)	0.143
Active filling velocity avg (mL/s)	128 (92–169)	104 (70–144)	130 (94–169)	0.610	0.011	0.76 (0.62–0.93)	0.008
Active filling velocity avg (%SV/s)	154 (106–205)	142 (94–222)	154 (107–204)	0.521	0.277	0.85 (0.65–1.12)	0.260
Active filling velocity peak (mL/s)	315 (222–456)	246 (176–382)	323 (227–464)	0.629	0.003	0.64 (0.47–0.85)	0.003
Vt_AI_—Volume Transient modes:							
Vt_AI_1	0.03 (− 0.45–0.54)	0.39 (− 00.2–0.66)	0.02 (− 0.47–0.51)	0.570	0.112	1.22 (0.96–1.56)	0.103
Vt_AI_2	− 0.08 (− 0.17–0.04)	0.00 (-00.1–0.13)	− 0.08 (-0.18–0.04)	0.598	< 0.001	1.27 (1.12–1.44)	< 0.001
Vt_AI_3	0.00 (− 0.22–0.22)	0.11 (-00.1–0.33)	− 0.01 (-0.22–0.21)	0.588	0.025	1.34 (1.05–1.71)	0.020
Vt_AI_5	− 0.02 (− 0.10–0.09)	0.06 (− 00.1–0.17)	− 0.02 (− 0.10–0.08)	0.611	< 0.001	1.35 (1.14–1.59)	< 0.001

Data presented as median (interquartile range). *P*-values were calculated for the comparison between patients with and without MACE. Univariate Cox regression analyses are presented as HR, Hazard ratios (95% confidence interval), and HR *P*-value, predictor significance. The predictive power of each biomarker is assessed via LDA and presented as median AUC 10-cross-fold validated, repeated for a hundred random data splits, AUC_k_. MACE indicates major adverse cardiac events; and avg, average. The passive and active filling metrics were not reported in those cases where diastasis was not found (diastasis = 0 s, n = 212).

## Results

### Patients

In total, 1021 patients (STEMI: n = 723; NSTEMI: n = 298) of the 1235 cohort were included in the study (No CMR: n = 126; incomplete protocol: n = 86; no follow-up: n = 2). Out of them, 73 patients suffered from MACEs (congestive heart failure: n = 20; reinfarction: n = 21; death: n = 32) ^23–25^. The population was predominantly male (74.5%) with a median age of 63 (IQR: 52–72) years, LVEF of 50.5% (IQR: 43.3–57.3) and IS of 13.4% of LV mass (IQR: 5.4–21.8). Their CMR and baseline clinical characteristics have been reported and discussed previously ^23–25^ and are reproduced in Supplemental Material IV.

### CMR-FT strain analysis (strain)

The CMR-FT strain results are described in Table 2. The MACE subgroup exhibited significantly lower global longitudinal, circumferential, and radial strains (*P* < 0.001) and a less uniform radial and circumferential contraction (*P* < 0.001).

### Automated volume transient AI analysis

#### LV segmentation and volume calculation

The automated segmentation of the SAx resulted in a median Dice score of 0.971 (IQR 0.945–0.981) for the LV cavity and 0.975 (IQR 0.959–0.982) for the LV epicardium. The correlation, r_s_, between the volumes calculated from the automated and manual segmentations were 0.916 and 0.919, for end-systolic and end-diastolic volume (ESV and EDV), respectively.

#### Volume transient conventional metrics (Vt)

Results are detailed in Table 2. The MACE subpopulation was characterized by a significantly shorter and more variable RR-interval (*P* < 0.001). Their systolic (*P* = 0.038), diastolic (*P* = 0.025), diastasis (*P* = 0.037) and time to diastasis (*P* = 0.013) was accordingly reduced. Nevertheless, only diastasis was significantly smaller when normalizing in time (*P* = 0.032). The passive (*P* = 0.002) and active (*P* < 0.001) contributions to LV filling were significantly compromised in MACE patients, as ESV and EF were significant (*P* < 0.001—See Table SIV.1), but not when normalizing by SV. Regarding velocities, the MACE cohort presented significantly lower systolic peak (*P* = 0.029), and active filling average (*P* = 0.008) and peak (*P* = 0.003) velocities. However, it was the passive filling average velocity the only one significant to MACE (*P* = 0.013) when normalizing by SV. Fig SIV.2 shows the average MACE and No MACE volume transients normalized by SV.

#### Volume transient AI ANALYSIS (Vt_AI_)

**Figure 4:**
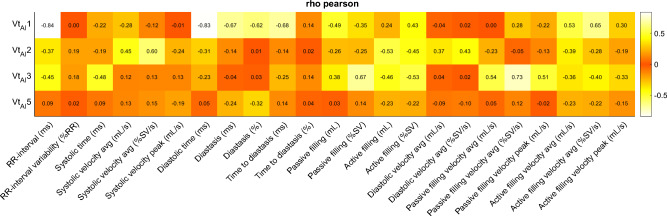
Correlation between AI-derived and conventional transient metrics. Heat map of Pearson correlation coefficients between AI-derived (columns) and conventional (rows) volume transient metrics.

95% of the variance was explained by the first 6 unsupervised modes of variation. Among them, modes 1, 2, 3 and 5 (Vt_AI_1, Vt_AI_2, Vt_AI_3 and Vt_AI_5) were determined the most relevant to MACE by the LDA stepwise analysis. These modes are interpreted as RR-interval (Vt_AI_1), systolic velocity (Vt_AI_2), passive vs active filling (Vt_AI_3) and diastasis (Vt_AI_5) (see Discussion). Figure 3 illustrates these modes and summarizes their significance, classification performance and distributions, stratified by MACE occurrence and infarct aetiology. The correlation between these AI metrics and the conventional transient ones is summarized in Fig. 4 (Pearson) and Supplemental Material VI (Spearman and R^2^).

### LV fully automated 3D shape analysis (LV_3D_)

The ES shape and 3D contraction descriptors were significant to MACE (*P* < 0.001) and proved superior to their corresponding stand‐alone versions ESV and LVEF^18^. Their statistics were reported in^18^, and are reproduced in Table SIV.1.

### Prognostic contributions

The results of the augmented prognostic contribution of proposed biomarkers to characterize LV contraction are shown in Table 3. Both LDA and Cox multivariable analysis converged to a nearly identical selection of variables for each of the models.

**Table 3:** Additional prognostic contribution of the proposed enhanced LV function characterization.

Model	Linear selection	AUC_k_	AUC_RS_	Cox selection	C_k_	C_RS_
LVEF	LVEF	0.683 (0.681—0.685)	0.687	LVEF	0.669 (0.668—0.670)	0.669
CMR	ESV, EDV	0.700 (0.698—0.702)	0.708	ESV, EDV	0.688 (0.687—0.690)	0.693
CMR + Vt	ESV, EDV, RR, t_diastolic_	0.708 (0.706—0.710)	0.721	ESV, EDV	0.688 (0.687—0.689)	0.693
CMR + Vt_AI_	ESV, EDV, Vt_AI_2, Vt_AI_3, Vt_AI_5	0.726 (0.723—0.730)	0.742	ESV, EDV, Vt_AI_3, Vt_AI_5	0.704 (0.702—0.707)	0.716
CMR + Strain	ESV, EDV, GLS	0.723 (0.721—0.725)	0.733	ESV, EDV, GLS	0.710 (0.708—0.711)	0.717
CMR + LV_3D_	ESV, EDV, C5, C16	0.738 (0.736—0.740)	0.750	ESV, EDV, C5, C16	0.728 (0.727—0.730)	0.736
ALL	ESV, EDV, Age, Killip	0.729 (0.727—0.733)	0.745	ESV, EDV, Age, Killip	0.714 (0.712—0.717)	0.726
ALL + Vt	ESV, Age, Killip, BSA, RR, t_diastolic_	0.737 (0.734—0.740)	0.759	ESV, Age, Killip, RR, v_diastolic_avg_	0.717 (0.714—0.721)	0.732
ALL + Vt_AI_	ESV, EDV, Age, Killip, Vt_AI_3, Vt_AI_5	0.746 (0.743—0.749)	0.769	ESV, EDV, Age, Killip, Vt_AI_3, Vt_AI_5	0.730 (0.727—0.732)	0.747
ALL + Strain	ESV, Age, Killip, BSA, Weight, Vessels, GLS	0.752 (0.749—0.754)	0.776	ESV, Age, Killip, BSA, GLS	0.732 (0.730—0.736)	0.748
ALL + LV_3D_	ESV, EDV, Age, Killip, C5, C16	0.747 (0.745—0.749)	0.766	ESV, EDV, Age, C5, C16	0.741 (0.739—0.743)	0.753
All + Vt_AI_ + Strain + LV_3D_	ESV, EDV, Age, C16, GLS, Vt_AI_3, Vt_AI_5	**0.774** (0.771—0.777)	**0.796**	ESV, EDV, Age, C16, GLS, Vt_AI_5	**0.760** (0.759—0.763)	**0.773**

Backward stepwise LDA and Cox results of additional prognostic metrics for MACE risk assessment. Considered features include conventional (Vt) and AI-derived (Vt_AI_) volume transient metrics, CMR-FT strains (strains), and LV 3D detail patterns (LV_3D_) descriptors, each on top of considering only CMR biomarkers (CMR) or the cardiovascular risk factors and patient characteristics of the study (ALL). A final experiment combines the 3 proposed directions of enhancement. Performance of LVEF is likewise included. The resulting significant selection of variables is reported along with performance, expressed as AUC and C-index (C) re-substitution (rs) and tenfold cross-validated (k), computed for a 100 random data splits and presented as median (interquartile range). All differences in AUC_k_ and C_k_ between models were statistically significant (*P* < 0.001), except for the ‘CMR’ and ‘CMR + Vt’ resulting Cox models. BSA indicates body surface area (m^2^); Killip, Killip class on admission; RR, RR-interval (ms); t_diastolic_, diastolic time (ms); v_diastolic_avg_, average diastolic velocity (mL/s); Vessel, number of diseased vessels.

Best performing results are highlighted in bold.

**Figure 5:**
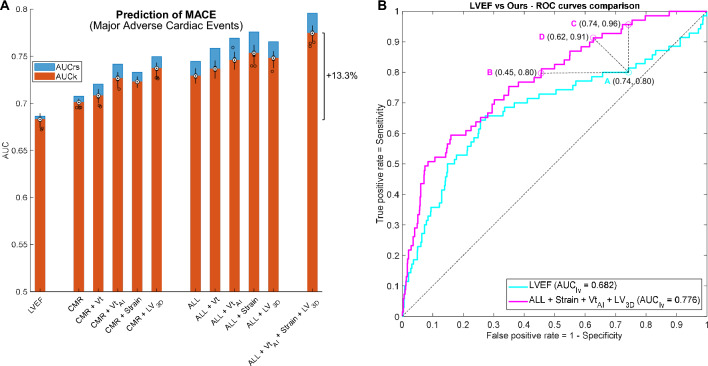
MACE prediction results and interpretation of the AUC differences. (**A**): MACE prediction performance results and prognostic contribution of the 3 proposed directions of enhancement. The performance of LVEF, the most established marker for AMI prognosis, is included as reference. Selection of significant variables is based on LDA multivariable stepwise analysis, and results are expressed in AUC resubstitution (rs, blue) and AUC 10-cross-fold validation (k, orange), repeated for a hundred random data splits (black distributions). All differences in performance were significant (*P* < 0.001). Note the remarkable improvement of the final configuration, combining the 3 proposed directions of enhancement. (**B**): Interpretation and implications of the AUC differences in ROC prediction curves, illustrated by the comparison between LVEF and our multivariable model, combining the 3 directions of enhancement. Given a recommended operating point of high sensitivity (80%) in the LVEF curve (point A), our model is able to predict MACE at the same sensitivity but reducing the false positive rate a 40% (point B), i. e., removing 379 false positive MACE predictions. Likewise, when operating at a similar false positive rate than LVEF (point C), our method improves sensitivity by 20%, detecting 96% of the MACE cases. Alternatively, the operating point D of our model could be chosen for MACE prediction, where both sensitivity and specificity are improved with respect to LVEF. This illustrates the potential for risk management improvement associated to superior AUC scores.

Prediction of MACE occurrence via conventional and AI automated volume transient metrics, strains and 3D LV markers proved significantly superior (*P* < 0.001) to LVEF using either CMR-derived (CMR: 2.49%; CMR + Vt: 3.66%; CMR + Vt_AI_: 6.3%; CMR + Strain: 5.86%; CMR + LV_3D_: 8.05% AUC improvement) or ALL clinical features (ALL: 6.73%; ALL + Vt: 7.91%; ALL + Vt_AI_: 9.22%; ALL + Strain: 10.1%; ALL + LV_3D_: 9.37% AUC improvement), as illustrated in Fig. 5A. Similar results were obtained in Cox analyses. The additional prognostic value was larger (*P* < 0.001) when using AI-derived metrics vs conventional transient ones (Table 3 and Fig. 5A).

The combination of the 3 directions of enhancement led to a major improvement compared to ALL baseline, and to the selection of one variable from each of these directions (GLS, C16 and Vt_AI_5 – and Vt_AI_3, in LDA). In comparison to LVEF, the AUC improvement was superior to 13% in both LDA and Cox analyses (See Fig. 5A and Table 3).

At a univariate level (see Table 2), GLS was the most prognostic variable (AUC_k_ = 0.710), followed by GCS, active filling contribution, and RR-interval. The AI-derived volume transient metrics were not among the most predictive variables, but ended up being the most complementary in multivariable settings.

## Discussion

This is the first large-sized multicenter CMR study to date that comprehensively characterizes the LV cardiac pump function after AMI using (I) LV volume temporal transient; and that combines it with (II) CMR-FT strain and (III) 3D shape analyses for improved risk assessment. We have successfully (a) developed a fully automated AI pipeline, available and easy to run, that segments the CMR SAx stacks and identifies the AMI acute contraction signatures associated to risk; (b) demonstrated the additional prognostic value of cardiac characterization via any of the 3 proposed directions of enhancement; and (c) combined these markers with the established CMR indexes, cardiovascular risk factors and patient characteristics towards a final CMR prediction model, that outdoes LVEF in a 13% (LDA, cross-validated AUC).

### Impact and clinical translation

While multiple trials have shown the incremental prognostic information of CMR-based risk models in AMI management, these models have not found their role in clinical practice^6,9,15^. The more common availability of echocardiography, the length of the CMR acquisitions, the requirement for manual interaction, and the increasing complexity of CMR multi-parameter models have been among the barriers for adoption^4,9^. Nevertheless, CMR post-infarction protocols have been shortened and CMR availability has significantly increased in recent years. Moreover, complex CMR prognostic predictors can be now synthetized into simple risk scores models^9^, and fully automated as shown in this study. Our work further facilitates the adoption of CMR post-infarction risk management by contributing to two of the pillars of healthcare systems: efficacy and efficiency^3^. That is, it leads to an improved prognostic assessment while reducing the analysis time.

Our automated pipeline for volume temporal transients runs on a standard laptop: it receives a CMR scan, segments the LV cavity, calculates the volume transient and summarizes the patient contraction dynamics into 4 biomarkers (Vt_AI_), that are used in the multivariable models to predict risk. The same applies to the automated shape analysis pipeline presented in^18^. This is done in the order of seconds, removing the burden of manual segmentations, and drastically reducing the analysis time. Moreover, the method is deterministic (i.e. given a scan, it always returns the same outputs) which, in turn, eliminates intra- and interobserver variability towards a more objective, standardized, and quantitative diagnosis. Conventional metrics extracted from volume transients often rely on rather qualitative detection landmarks (e.g. diastasis), which may be problematic especially in pathological cases. Importantly, our proposed Vt_AI_ markers are robust to outliers and abnormal cases. Furthermore, the strong correlation between the automated and manual volumes, along with the high values and small variance of the segmentations dice scores (see Results), supports the accuracy and precision of the automated pipeline, which additionally outdoes commercially available software in performance^41^.

LV contraction dynamics are conventionally based on global function metrics evaluated at two single time points (ES and ED, or their ratio, LVEF). The tree proposed approaches to unveil further prognostic value complement each other in the final multi-variate regression model: first, the temporal resolution is increased by the volume transient metrics (from two points to the whole cycle); second, the bulk tissue contraction is assessed by the strain patterns (CMR-FT—from global function to quantification in longitudinal, radial and circumferential directions); and third, the 3D anatomical detail is studied by statistical shape models^18^. The relevance of looking at AMI management from all these different angles is evidenced by the results of the final risk-assessment model that combines global function descriptors (ESV, EDV), 3D contraction patterns (C16), strains (GLS), contraction dynamics indexes (Vt_AI_3, Vt_AI_5) and patient characteristics (age).

Our model provides a remarkable boost in MACE prediction: 13% AUC improvement, when comparing against LVEF (Fig. 5A and Table 3), the reference marker according to AMI guidelines^4,6^. The implications of these AUC improvements, in terms of specificity and sensitivity, are illustrated in Fig. 5B. The large size of the cohort of this study, the small gap between resubstitution and cross-validation metrics, and the narrow variance when using 100 random data splits (see Fig. 5A and Table 3) suggest the robustness of our method for MACE prediction.

The prognostic contribution of the proposed enhanced characterization of cardiac contraction is in line with recent publications exploring alternative characterizations to LVEF^42,43^. For example, Dong et al. demonstrated the contribution of CURE and RURE to AMI risk stratification in a cohort of 450 STEMI patients^42^; and Lange et al. proved the incremental prognostic value of an improved myocardial deformation assessment above LVEF (a c-index increase from 0.7 to 0.74, *p* = 0.03) in a trial including 566 STEMI patients^43^. Our work incorporates most of the prognostic parameters discussed in these publications; considers a larger cohort of patients, including both STEMI and NSTEMI aetiologies; and results in a superior prognostic contribution in comparison to LVEF (a c-index increase from 0.67 to 0.76, *p* < *0.001*—See Table 3).

As a result of the automated analysis, we have built a reference LV volume transient atlas from 1000 + AMI subjects that captures the average post-infarct contraction behavior along with the main variations (PCA modes). The atlas and the associated methods to calculate the herein proposed Vt_AI_ markers and the resulting risk models, made publicly available (doi.org/10.6084/m9.figshare.16735300), will not only allow the validation of the presented findings but also further AMI studies, computer simulations, and synthetic patient data generation for training algorithms or educational use, among others. Moreover, while the AI decomposition methodology has been deployed using CMR volume transients, its application to echocardiography is straightforward. The segmentation algorithm, extendable to different protocols and scanners to the ones used in this study as well as to other cardiac diseases^44^, is likewise available.

### Interpretability: unravelling the AMI contraction

While infarct severity is associated with functional and morphological alterations, and certain acute changes have proven significant to AMI prognosis, their interplay in modulating risk remains unsolved^6,13,45,46^. In this regard, we have identified detailed contraction traits, combining strains, LV shape and volume transient, significantly relevant to MACE occurrence.

Conventional descriptors of the contraction dynamics have identified the RR-interval length and the active and passive contributions to filling, in mL, as particularly related to MACE occurrence (see Table 2). Since patients at risk exhibit a decreased LVEF^4,6^, their passive and active contributions are accordingly reduced and, in consequence, their RR-interval shortened to increase cardiac output^47^. This, in turn, leads to a smaller preload and a less effective contraction, with the corresponding drop in LVEF (negative feedback loop).

RR variability was also found to be highly prognostic. It can be hypothesized that this beat-to-beat variation could be related to atrial fibrillation, as evidenced in^48^, especially given the mentioned fast heart rates, related to ventricular tachycardia and fibrillation risk according to the MADIT-ICD score^49^, that characterize our MACE population. Nevertheless, and oppositely, the RR variability has been shown beneficial in other atrial fibrillation disease models^50^.

One of the advantages of our AI methodology is that the resulting Vt_AI_ variables are not obscure features for prediction improvement, but they rather encode explainable contraction patterns, as shown in Fig. 3. Thus, Vt_AI_1 can be primarily interpreted as RR-interval length (r_p_ = -0.84); Vt_AI_2, as average systolic velocity (r_p_ = 0.60); Vt_AI_3, as a balance between the active and passive contributions (passive filling: r_p_ = 0.67); and Vt_AI_5, as diastasis once corrected for RR-interval length (diastasis: r_p_ = -0.32; r_s_ = -0.43. See Supplemental Material VI). Nevertheless, these contraction variations contain more information than just a single conventional metric, reason why they are also correlated to other variables (i.e. in a contraction with reduced RR-interval the diastolic and diastasis times will be likewise compromised; therefore, Vt_AI_1, correlated to RR-interval, is also correlated to these indexes) and reason why they led to greater prognostic contributions.

In this context, the minmax normalization prior to the AI analysis, standardizing the transients by removing ventricle size and SV information, should be considered when visually interpreting the results (See Supplemental Material II). This normalization decreases the univariate predictive power of the Vt_AI_ markers to the benefit of removing cofounding factors and facilitating their contribution to multivariable settings. This is evidenced by their significant contribution in any of the considered multivariable approaches (Table 3), unlike, for instance, the CMR tissue markers (MVO and IS), which are predictive according to the univariate analysis but do not significantly add value to the multivariable models. Vt_AI_3 and Vt_AI_5, both interpreted as related to diastolic function, are the AI-derived transient markers that contribute the most towards additional prognosis in the multivariable settings (Table 3). While mechanistic explanations are to be further explored, a plausible interpretation is that, in patients at risk, RR-interval shortening compromises ventricular relaxation. This may come at the expense of diastasis (Vt_AI_5), and/or faster dynamics with a swift in the relative filling contributions (%SV) towards the passive phase (Vt_AI_3). This in turn explains the consequent significant increase in normalized passive filling velocity (note that the actual passive filling velocities, in mL/s, without normalization, are smaller for the MACE population, due to a smaller LVEF—see Table 2). This is particularly relevant for the STEMI population (see Fig. 3).

Likewise, the 3D LV patterns, already reported and interpreted in^18^, demonstrated being consistent with ESV, LFEF and ES myocardial thickness^45,46^ and highlighted the additional prognostic value of a lateral and a basal contraction impairment. The interplay between these myocardial 3D shape changes and micro-damage, in the form of IS and MVO, was also evidenced.

Strain has been proven an important parameter to evaluate risk after an infarct, as discussed in the introduction section. This is evidenced in this work, where all the CMR-FT strain metrics reported were highly significant to MACE occurrence (Table 2). The compromised GLS, GRS and GCS that the MACE population exhibits are in harmony with the LVEF decrease associated to risk^4,6^. Besides, the observed relationship between risk and impaired contraction uniformity (CURE and RURE) is widely discussed in^9^. The prognostic value of GLS, superior to LVEF and incremental to the baseline markers, is in accordance with^21^, that argues for its adoption in clinical practice.

The proposed risk models have superior prognostic value for patients with decreased LV function (LVEF < 35%), identified as high-risk group, in accordance with the literature^4,10^ (Fig. SV.2). With respect to infarct aetiology, the risk models that include CMR-FT strain or 3D LV patterns are more predictive for the NSTEMI population, despite the study being biased to STEMI patients (see Fig. SV.3 and Results—Patients). The final model, that combines strains, AI transient metrics and 3D LV metrics, reports a similar performance for STEMI (AUC = 0.795) and NTEMI (AUC = 0.799), and, thus, clinical validity for both infarct types could be claimed. If analyzed separately, both systolic and diastolic components significantly contribute to the multivariable risk assessment models (see Supplemental Material VII). However, as expected, the combination of systolic and diastolic function, that is, the entire transient, is more prognostic than each of the components on its own, especially in the systolic scenario (in line with the fact that the modes that provide more additional prognostic value, Vt_AI_3 and Vt_AI_5, are related to diastolic function, as mentioned above).

To sum up, patients at risk of MACE occurrence have a compromised cardiac function, which contributes to an impaired global contraction (decreased LVEF and GLS) and, therefore, to a reduced RR-interval, in order to maintain the cardiac output. This, in turn, leads to diastolic impairment, exhibited in the form of compromised diastasis and active filling.

### Limitations

Patients were imaged within 10 days after infarction in the absence of optimal post-infarction CMR imaging time recommendations^9^. The effect of this post-infarction imaging time on the proposed AI-derived metrics has not been assessed. It is however hypothesized that we are capturing acute injury, whose extent and phenotype can predict outcome, and therefore long-term LV adaptions. The main objective of this work was to evaluate this hypothesis in a clinical cohort with the proposed enhanced characterization of cardiac contraction, and not to optimize MACE prediction. This motivates the herein explained identification of metrics based on linear models that eases interpretability. Future research could explore the use of advanced nonlinear classifiers, as illustrated in^51^, to improve performance in MACE prediction at the cost of losing the ability to interpret findings. CMR long-axis views were excluded for simplicity, but ideally could be incorporated to correct for SAx misalignment and improve the cavity volume calculations. Likewise, while only CMR-FT global strains and uniformity ratio estimates have proved prognostic in the literature^7,9,13,19,20^, future work could consider a more detailed strain analysis, in time and by regions, especially given the outstanding prognostic power of GLS. Similar methodologies to the PCA analysis formulated in this study could be considered for this. The resolution of the volume transients herein derived is limited to the resolution of the CMR scan. Given the limited number of MACE events (class imbalance), splitting the cohort into an independent testing dataset would be rather noisy. The combination of cross-fold validation, repeated for a hundred data-splits, is therefore preferred as it ensures fair performance and robust conclusions reporting, as argued in^36,40^. The study population is biased towards male sex. Finally, the results are limited to AMI patients that can undergo a CMR scan. This is an active field of research and future investigations are required to integrate these measures into standard clinical practice.

## Conclusion

This large multicenter study evidences the prognostic value of a comprehensive characterization of cardiac contraction by the novel use of LV volume transients, combined with metrics of strain and 3D shape, in post-infarction management. Moreover, it further contributes towards the adoption of CMR multivariable models in clinical practice by demonstrating the feasibility of fully automated analysis and the relevance of these models in AMI stratification.

### Supplementary Information

Supplementary Information 1.

Supplementary Video 1.

Supplementary Video 2.

## Data Availability

The data that supports the findings is available upon reasonable request to the corresponding author of this manuscript.

## Abbreviations

2D: Two-dimensional
3D: Three-dimensional
AI: Artificial intelligence
AMI: Acute myocardial infarction
AUC: Area under the receiver-operator characteristic curve
C-index: Concordance index
CMR: Cardiac magnetic resonance
CMR-FT: CMR myocardial feature tracking
CURE: Circumferential uniformity ratio estimate
ED: End-diastole
EDV: End-diastolic volume
ES: End-systole
ESV: End-systolic volume
FT: Feature tracking
GCS: Global circumferential strain
GLS: Global longitudinal strain
GRS: Global radial strain
IQR: Interquartile range
IS: Infarct size
LDA: Linear discriminant analysis
LGE: Late gadolinium enhancement
LV: Left ventricle
LV_3D_: LV 3D patterns markers
LVEF: Left ventricular ejection fraction
MACE: Major adverse cardiac events
MVO: Microvascular obstruction
NSTEMI: Non-ST‐segment-elevation myocardial infarction
PCA: Principal component analysis
r_p_: Pearson correlation coefficient
r_s_: Spearman correlation coefficient
RURE: Radial uniformity ratio estimate
SAx: Short-axis stack
STEMI: ST‐segment-elevation myocardial infarction
SV: Stroke volume
Vt: Conventional volume temporal transients
Vt_AI_: AI-derived volume temporal transients
