# Supplementary material for: Comprehensive characterization of cardiac contraction for improved post-infarction risk assessment
Source: Sci Rep. 2024 Apr 18;14:8951. doi: 10.1038/s41598-024-59114-3 (PMC11026383; doi:10.1038/s41598-024-59114-3)
Supplement: Supplementary file 1 — Supplementary Information 1. [file 41598_2024_59114_MOESM1_ESM.pdf]

SUPPLEMENTAL MATERIAL

Comprehensive Characterization of Cardiac Contraction for Improved Post-Infarction Risk Assessment

Jorge Corral Acero, Pablo Lamata, Ingo Eitel, Ernesto Zacur, Ruben Evertz, Torben Lange, Sören J. Backhaus, Thomas Stiermaier, Holger Thiele, Alfonso Bueno-Orovio, Andreas Schuster, Vicente Grau.

Table of Contents

SUPPLEMENTAL MATERIAL I – LV Segmentation 2
SUPPLEMENTAL MATERIAL II – Volume Transient Normalization 4
SUPPLEMENTAL MATERIAL III – Dimensionality Reduction 5
SUPPLEMENTAL MATERIAL IV – Additional Patient Characteristics 8
SUPPLEMENTAL MATERIAL V – Endpoint Prediction Additional Results 11
SUPPLEMENTAL MATERIAL VI – Modes Correlation Additional Results 14
SUPPLEMENTAL MATERIAL VII – Systolic vs Diastolic Components 15
SUPPLEMENTAL MATERIAL VIII – TRIPOD and STROBE Checklists 17

SUPPLEMENTAL MATERIAL I – LV Segmentation

A 2-step state-of-art approach was applied to segment the LV endo- and epicardium (see Fig. SI.1), from which the LV cavity and myocardium can be estimated (32,33):

- In the first step, pre-processing, the images are re-oriented, cropped and normalized. Considering the mid-ventricular SAx slice as reference, the first neural network (NN) detects the position of the heart and defines a region of interest (ROI) of 139.7x139.7 mm centered in the LV. Based on these LV centroid and ROI, the LV is aligned to a canonical position by a rigid registration of the same SAx reference slice to the atlas built in (33). The SAx is then cropped accordingly, and the intensities are normalized.
- The second step, fine segmentation, applies another NN to the pre-processed images to regress the enhanced LV segmentation. Fig. SI.2 illustrates the architecture of this fine-segmentation network. A final postprocessing is applied to binarized the segmentation predictions and improve the segmentation quality.

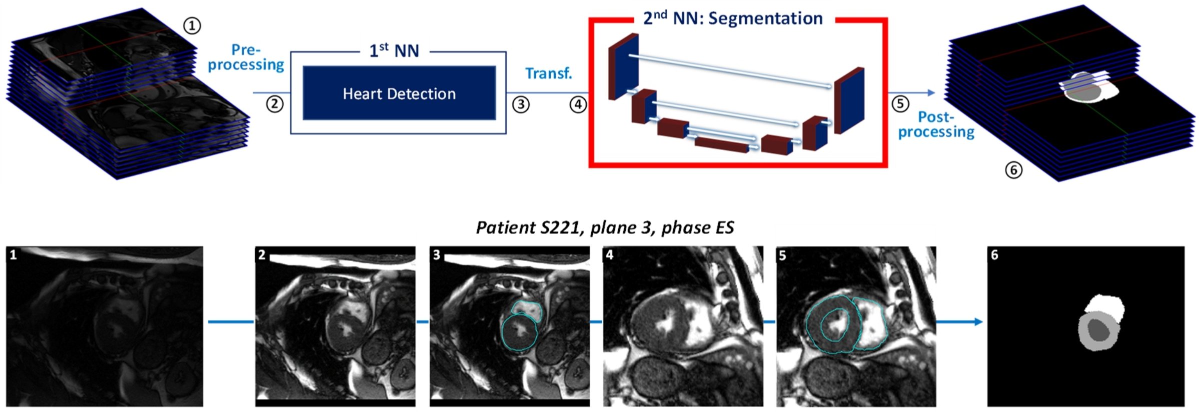

Fig. SI.1 – Scheme of the proposed 2-step segmentation pipeline (top) along with a step-by-step explanatory illustration of its application on patient S221 (bottom). This pipeline design addresses canonical orientation for LV regional metrics quantification and label imbalance for segmentation performance improvement. See (33) for further details.

A cohort of 100 patients of the study, manually segmented ,with random resolutions and endpoints, was used to train the 2 NNs, following a 5-fold cross-validation strategy and using the same training-validation-testing split ratio as in (33). Translation, rotation and flipping were used for augmentation. Architectures and implementation are detailed in (32,33). Segmentation performance assessment is based on endocardium and epicardium gold standard Dice scores. This metric accounts for the overlap between manual segmentation and automated prediction and varies between 0 and 1, with 1 corresponding to a perfect match. Fig. SI.3 provides a visual sample of the segmentation results.

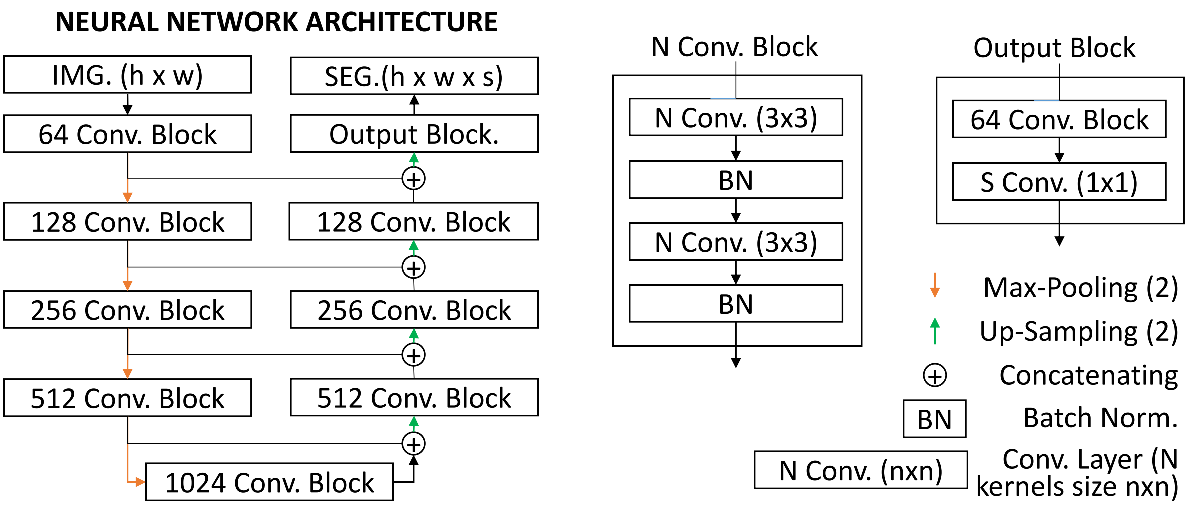

Fig. SI.2 – Graphical overview of the convolutional neural network structure, skip connections following a U-Net architecture, that is applied to achieve the fine-segmentation (2nd pipeline step). Reproduced from (32).

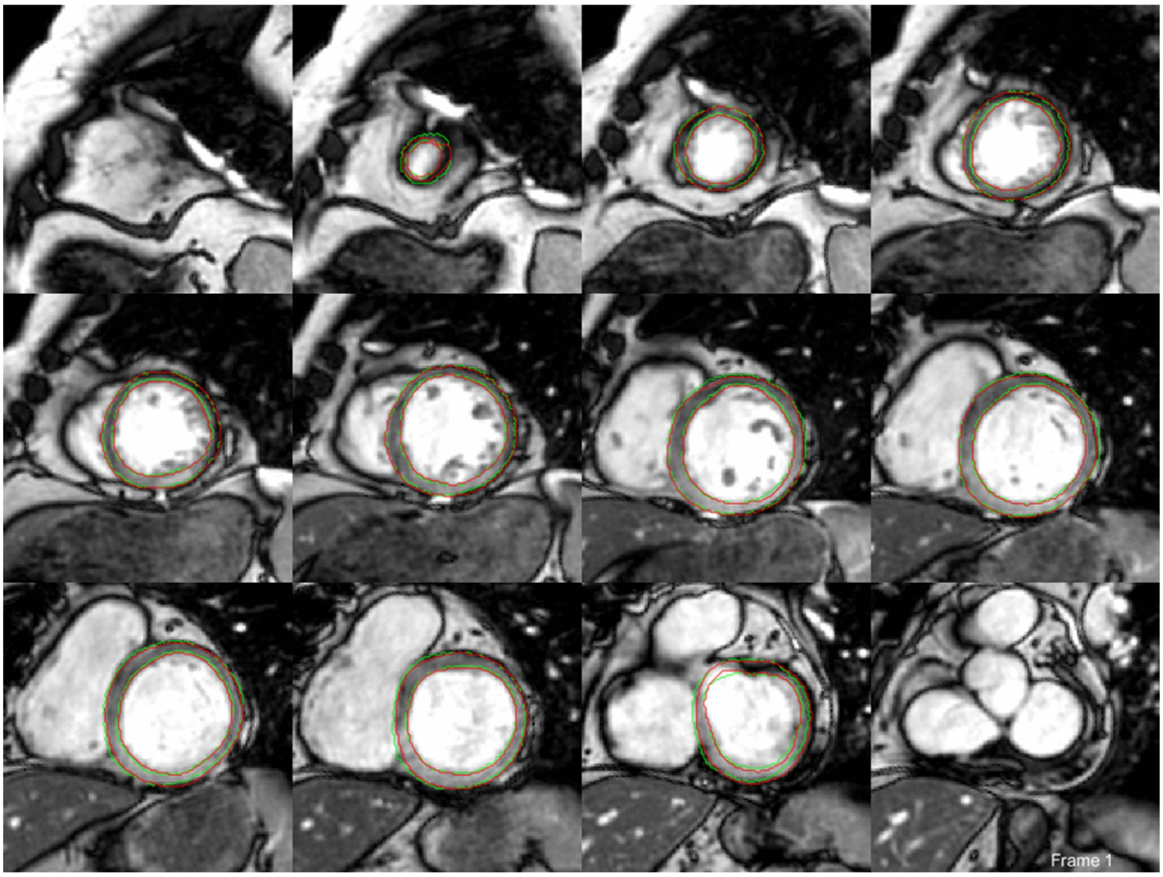

Fig. SI.3 – Segmentation results of a representative patient (median Dice) at ED. The green contours correspond to the LV reference segmentation (manual segmentation); and the red contours, to the prediction results based on our proposed 2-step deep learning approach.

SUPPLEMENTAL MATERIAL II – Volume Transient Normalization

The LV volume temporal transients are described by the magnitude of the curve (i.e. maximum and minimum LV volumes) and by its shape (i.e. relative filling contributions, presence or absence of diastasis, etc.). While the latter rather involves subtle changes and its understanding is the aim of this study, the former, that contains information about ventricle size and amplitude of contraction or stroke volume, is a major source of variability and can be completely described by the well-established EDV, ESV and LVEF markers. This motivates the standardization in magnitude, to remove cofounding factors, towards facilitating multivariate models, and variability noise, so that the PCA analysis concentrates on these subtle transient shape changes. Thus, a standard minmax normalization is proposed:

$$\bar{V}(t) = \frac{V(t) - ESV}{SV},$$

where $V(t)$ represents the original LV volume transient (mL/s); $\bar{V}(t)$, the normalized LV volume transient (%/s); ESV, the end-systolic volume (mL); and SV, the stroke volume (mL), calculated as end-systolic minus end-diastolic volume.

Fig. SII.1 illustrates two LV volume temporal transients from patients of a similar ventricle size before and after the proposed normalization. The intra-relationships within a transient are preserved, that is, the active versus passive contribution balance or the systolic and diastolic velocities ratio, for instance, are constant before and after normalization. However, the volumes are no longer absolute values but expressed as percentage of SV, enabling for direct inter-comparison between patients of a very different ventricle size or LVEF. This should be considered when drawing conclusions on the resulting transient patterns, presented in the main manuscript (see Fig. 3). Thus, a plausible conclusion from Fig. SII.1 is not that the passive filling is faster in patient 1 but that, considering the rest of the transient velocities, the passive filling is relatively faster in patient 1.

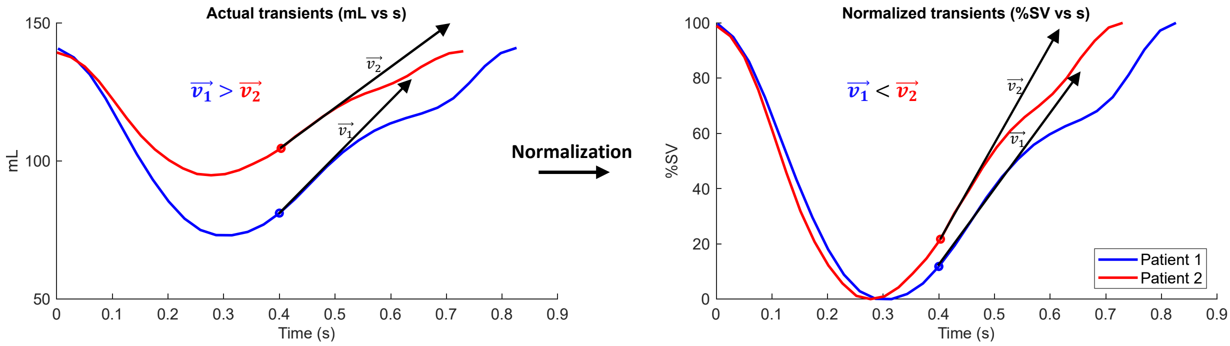

Fig. SII.1 – LV volume temporal transient normalization for 2 patients. **LEFT**: LV volume temporal transients expressed in absolute volumes (mL/s). **RIGHT**: Normalized LV Volume temporal transients (%SV/s). The normalization standardizes for ventricle size and stroke volume, enabling for transient 'shape' inter-comparisons. In consequence, the passive filling peak velocity, considering its smaller SV and the rest of the transient velocities, is relatively faster for patient 2 ($\vec{v_2} > \vec{v_1}$), while, in absolute terms, this is the opposite ($\vec{v_1} > \vec{v_2}$).

SUPPLEMENTAL MATERIAL III – Dimensionality Reduction

The LV volume temporal transients are obtained by LV cavity volume integration in each frame of the cardiac cycle, as explained in the manuscript (see Methods). Therefore, the transients are described as a collection of 2D points, where the 'x' dimension corresponds to the trigger time of the frame, or time-point within the cardiac cycle, and the 'y' dimension represents the integrated cavity volume. Given the multicenter and multi-scanner nature of the study, patients are imaged at different resolutions, which results in 2D collection of points of different sizes (i.e. from 20 frames, and, in consequence, 20 pairs of time and volume 2D points, to 50 frames). Thus, a first step resamples the obtained volume transients, via splines, to ensure that all of them are described with the same number of points, that is, 30 points (evenly distributed in the 'x' or time dimension). This effectively means that the LV volume transient of each patient is described by 60 variables (30 points x 2 dimensions), which take a certain different value per patient. A second step normalizes the 'y' or volume dimension, as explained in Supplemental Material II, to standardize in ventricle size and SV.

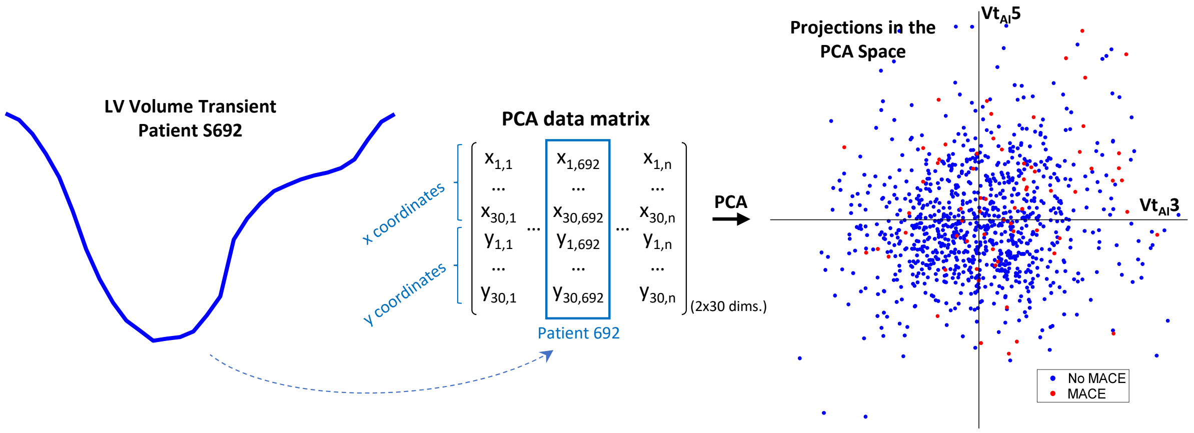

Fig. SIII.1 – **LEFT**: LV Volume transient of patient 692. **MIDDLE**: The 60 variables that describe the patient volume transient are sorted in columns, one per patient, to apply PCA. **RIGHT**: Projection of the patients in the 2 PCA directions that contributed the most to multivariate models (see Table 3) in order to maximize MACE (red) vs No MACE (blue) differences, that it, $Vt_{AI}3$ and $Vt_{AI}5$. We moved from 60 to just 2 transient variables per patient, which, additionally, are interpretable.

PCA, the dimensionality reduction technique applied in this work, is able to encode the information contained in these 60 variables (LV volume transient variability) into a few variables, the PCA modes, that describe the main LV volume transient variations. Mathematically, this is done by finding the vector space whose basis are the orthogonal directions that maximize the variance of the data, and subsequently projecting the data in this new space (See Fig. SIII.1).These directions that maximize the variance are, precisely, the PCA modes of variation that represent the way in which the LV volume transient varies in the population (i.e. RR-interval length, passive vs active filling relative contributions, etc.). As illustrated in Fig. SIII.2, if we move in the direction of a mode, we can see how the mean transient curve deforms in its particular way. This allows to describe each LV volume transient as a mean volume transient curve plus the variations encoded by each linear anatomical mode times the amount and direction of variation, or PCA coefficients (See Fig. SIII.4):

$$LV\ Volume\ transient = \varphi_0 + \sum_i Vt_{AI}i \cdot \varphi_i,$$

where φ_0 represents the mean volume transient, φ_i the anatomical PCA modes, and $Vt_{AI}i$ their respective PCA coefficients. Each of the modes, i, is therefore a continuous variable, that accounts for

a particular shape variation, which has a certain value for each patient, $Vt_{AI}i$, and whose MACE predictive power can be analyzed. In other words, we can investigate which LV transient features or contraction patterns (modes) are related to AMI prognosis.

The modes are sorted in descending order of importance according to the amount of variability of the population that they explain (i.e. in our results 51.6% variability is described by mode 1; 18.4%, by mode 2; etc.). As we progressively incorporate modes, the LV transient reconstruction improves to the point that with only few modes we can accurately approximate any volume transient (i.e. 95.99% of LV transient variance described by first 6 modes), and hence the dimensionality reduction is achieved (See Fig. SIII.3).

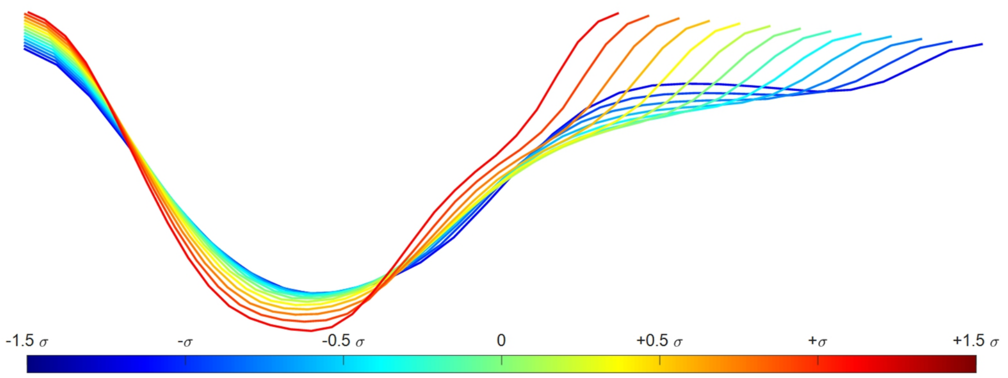

Fig. SIII.2 – The PCA direction of maximum variability corresponds to mode 1. In our results, the shape variation that it encodes is interpreted as mainly RR-interval length, although some other subsequent changes in transient morphology (i.e. diastasis) can be appreciated. As we move along the direction of mode 1, the RR-interval of the mean LV volume transient is reduced (positive $Vt_{AI}1$) or increased (negative $Vt_{AI}1$), proportionally to the value of its PCA coefficient $Vt_{AI}1$.

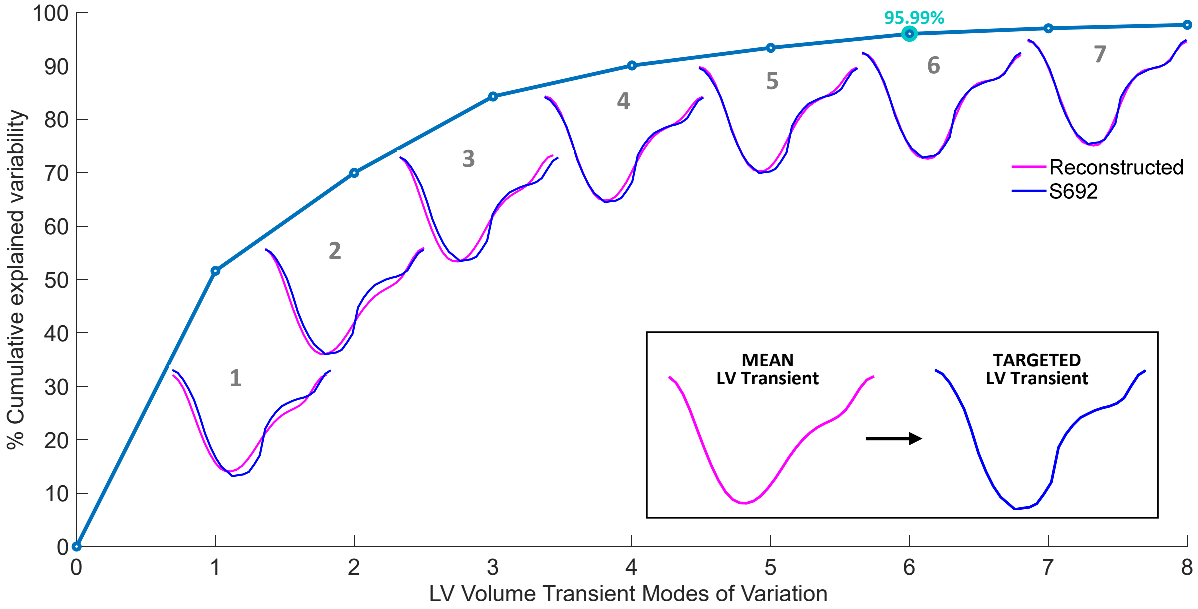

Fig. SIII.3 – Cumulative % of the population variance explained by the modes. As we incorporate more modes, we account for more variance and we accurately approximate the target shape (Patient 692). The first few modes account for the majority of the variance, and as we move to latter modes the improvement in reconstruction is smaller (i.e. mode 6 vs mode 7).

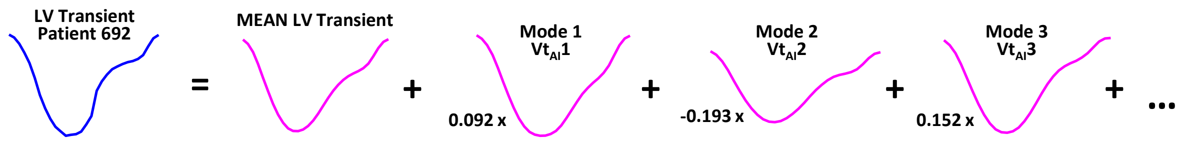

Fig. SIII.4 – Following PCA application, each LV volume temporal transient is decomposed into the mean volume transient (average of population of LV transients) plus the anatomical modes (transient variations, illustrated here as mean plus the positive extreme) times the corresponding PCA coefficient, $Vt_{AI}i$. The figure illustrates the decomposition of patient 692.

SUPPLEMENTAL MATERIAL IV – Additional Patient Characteristics

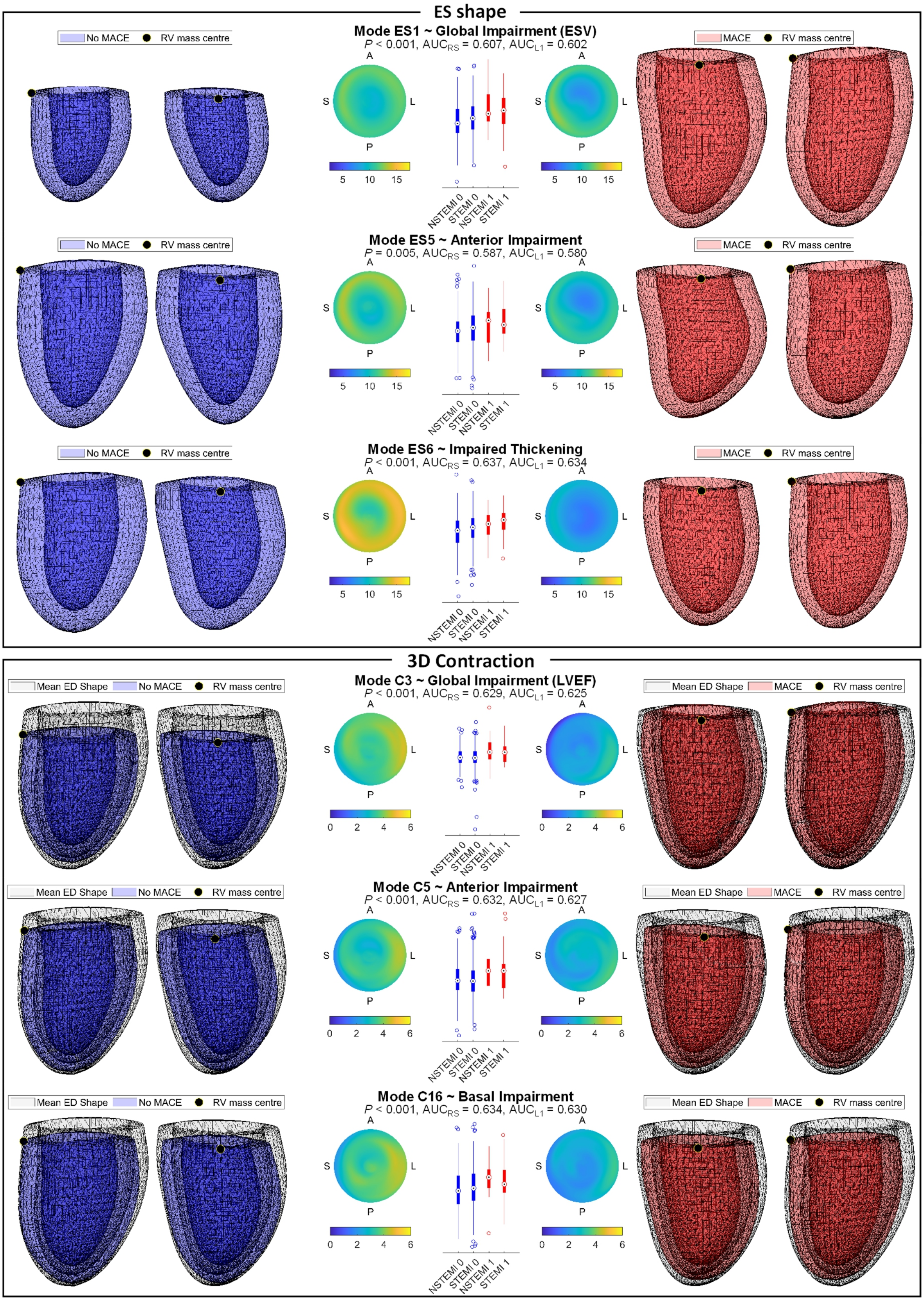

Fig. SIV.1 – The 3 main ES shape features (ES1, ES5, ES6) and contraction patterns (C3, C5, C16) related to MACE occurrence, resulting from the shape analysis explained in detail in (18), are

illustrated. Meshes shown in anterior and septal views, and as differential thickness maps (ED-ES thickness) on polar plots of the AHA model. To facilitate comparisons, the contractions are applied on the mean ED shape (reference transparent surface) and visualized as resulting ES shapes. No MACE (blue, class 0) and MACE (red, class 1) representations correspond to the 5th and 95th percentiles in the LDA direction. *P* values and both resubstitution (RS) and leave-one-out (L1) AUCs shown along MACE and no MACE distributions, further stratified into infarct aetiology (STEMI and NSTEMI). Reproduced from (18).

Table SIV.1 – Basic patient characteristics, cardiovascular risk factors and 3D LV biomarkers

Variable	ALL Patients	MACE (n = 73)	No MACE (n = 948)	AUC_k	*P* value	HR	HR *P*-val
Age	63 (52 - 72)	72 (61 - 77)	63 (52 - 72)	0.659	<0.001	1.80 (1.39 - 2.32)	<0.001
Sex	753/1011 (74.5)	46/71 (64.8)	707/940 (75.2)	0.505	0.052	0.81 (0.65 - 1.00)	0.050
Height, cm	2 (1 - 2)	2 (1 - 3)	1 (1 - 2)	0.597	0.003	0.69 (0.55 - 0.86)	0.001
Weight, kg	81 (72 - 90)	76 (70 - 86)	82 (73 - 90)	0.568	0.035	0.82 (0.64 - 1.05)	0.110
Cardiovascular risk factors							
Current smoking	405/935 (43.3)	19/63 (30.2)	386/872 (44.3)	0.522	0.029	0.75 (0.57 - 0.97)	0.032
Hypertension	716/1010 (70.9)	61/71 (85.9)	655/939 (69.8)	0.545	0.004	1.53 (1.13 - 2.08)	0.006
Hyperlipoproteinemia	624/1005 (62.1)	45/71 (63.4)	579/934 (62.0)	<0.5	0.816	1.03 (0.81 - 1.30)	0.824
Diabetes mellitus	231/1010 (22.9)	26/71 (36.6)	205/939 (21.8)	0.526	0.004	1.34 (1.09 - 1.64)	0.005
Body mass index, kg/m^2	27.4 (25.0 - 30.4)	27.0 (25.2 - 30.8)	27.4 (25.0 - 30.3)	<0.5	0.959	1.03 (0.82 - 1.30)	0.773
Body surface area, m2	1.95 (1.83 - 2.08)	1.88 (1.76 - 2.00)	1.96 (1.83 - 2.08)	0.593	0.006	0.74 (0.59 - 0.94)	0.014
Killip class on admission				0.573	<0.001	0.52 (0.41-0.65)	<0.001
1	899/1011 (88.9)	49/71 (69.0)	850/940 (90.4)				
2	76/1011 (7.5)	13/71 (18.3)	63/940 (6.7)				
3	20/1011 (2.0)	4/71 (5.6)	16/940 (1.7)				
4	16/1011 (1.6)	5/71 (7.0)	11/940 (1.2)				
Nr. of diseased vessels				0.567	0.003	1.40 (1.12 - 1.75)	0.003
1	502/1011 (49.7)	25/71 (35.2)	477/940 (50.7)				
2	310/1011 (30.7)	23/71 (32.4)	287/940 (30.5)				
3	199/1011 (19.7)	23/71 (32.4)	176/940 (18.7)				
TIMI flow grade post-PCI				<0.5	0.318	0.95 (0.77 - 1.16)	0.598
0	19/1011 (1.9)	1/71 (1.4)	18/940 (1.9)				
1	21/1011 (2.1)	2/71 (2.8)	19/940 (2.0)				
2	78/1011 (7.7)	8/71 (11.3)	70/940 (7.4)				
3	893/1011 (88.3)	60/71 (84.5)	833/940 (88.6)				
CMR biomarkers							
LV ESV, mL	70 (53 - 91)	86 (60 - 110)	69 (53 - 90)	0.599	0.004	1.43 (1.18 - 1.73)	<0.001
LV EDV, mL	144 (117 - 171)	145 (121 - 170)	144 (117 - 172)	<0.5	0.987	1.05 (0.83 - 1.33)	0.679
LVEF (%)	50.5 (43.3 - 57.3)	40.6 (33.1 - 52.2)	50.8 (44.0 - 57.5)	0.683	<0.001	0.80 (0.74 - 0.87)	<0.001
Infarct size, mL	17.2 (6.4 - 30.2)	24.6 (9.7 - 36.4)	16.7 (6.0 - 29.9)	0.591	0.006	1.29 (1.08 - 1.53)	0.005
Infarct size (% LV mass)	13.4 (5.4 - 21.8)	20.3 (9.6 - 28.9)	13.1 (5.3 - 21.4)	0.609	0.001	1.44 (1.18 - 1.76)	<0.001
MVO, mL	0.00 (0.00 - 1.90)	0.40 (0.00 - 3.00)	0.00 (0.00 - 1.80)	0.543	0.060	1.27 (1.11 - 1.45)	<0.001
MVO (% LV mass)	0.00 (0.00 - 1.39)	0.32 (0.00 - 2.15)	0.00 (0.00 - 1.27)	0.547	0.044	1.26 (1.09 - 1.46)	0.002
ES Shape				0.680			
Mode 1	-5 (-135 - 126)	62 (-61 - 233)	-10 (-136 - 119)		0.002	1.49 (1.18 - 1.87)	<0.001
Mode 5	0 (-34 - 36)	-11 (-51 - 22)	1 (-33 - 37)		0.014	0.73 (0.58 - 0.91)	0.006
Mode 6	-3 (-30 - 28)	-21 (-44 - 3)	-2 (-29 - 30)		<0.001	0.64 (0.50 - 0.81)	<0.001
Contraction Displacement				0.716			
Mode 3	-4 (-54 - 62)	49 (-29 - 106)	-6 (-55 - 59)		<0.001	1.70 (1.36 - 2.12)	<0.001
Mode 5	3 (-37 - 39)	-30 (-57 - 23)	4 (-33 - 40)		<0.001	0.63 (0.51 - 0.78)	<0.001
Mode 16	1 (-15 - 14)	-9 (-19 - 5)	1 (-14 - 15)		<0.001	0.65 (0.52 - 0.82)	<0.001

Data presented as n/N (%) or median (interquartile range). *P* values calculated between MACE/No-MACE groups. Hazard ratios (HR) presented with 95% confidence intervals and predictor significance. AUC_k provides the predictive power of each biomarker, assessed via LDA (median AUC, 10-cross-fold validated, 100 random data splits). MACE: major adverse cardiac events; PCI: percutaneous coronary intervention; TIMI: Thrombolysis in Myocardial Infarction. Reproduced from (18).

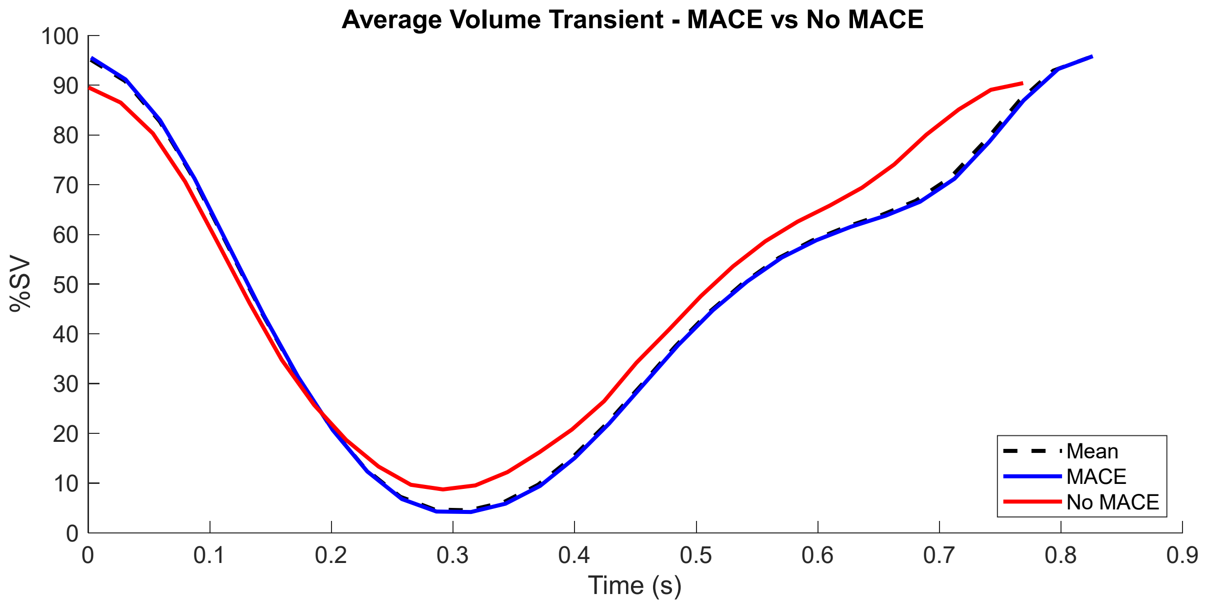

Fig. SIV.2 – Average LV volume transient, normalized by stroke volume, of the AMI cohort (dashed line), along with the average transient stratifying by MACE (red) and no MACE (blue).

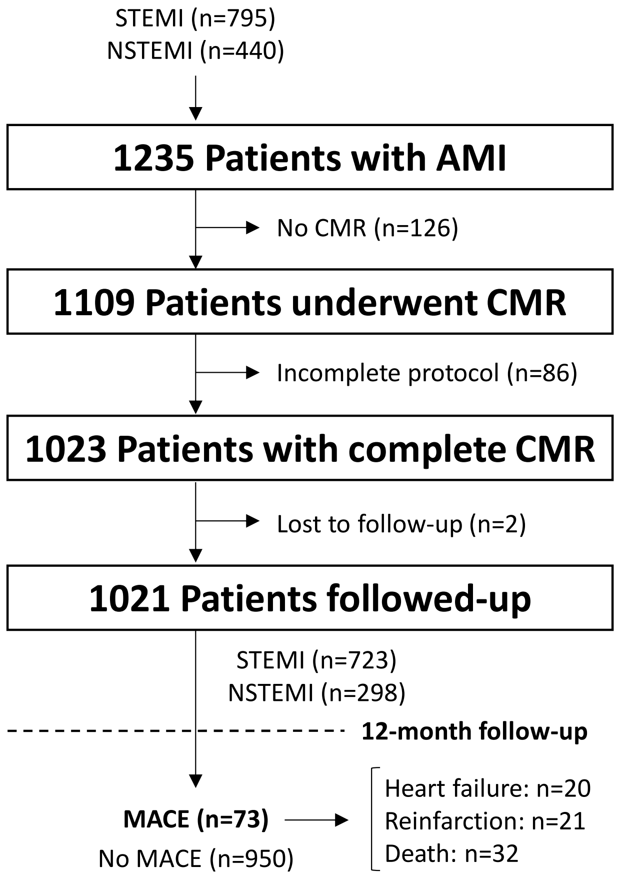

Fig. SIV.3 – Study flowchart. AMI indicates acute myocardial infarction; CMR, cardiac magnetic resonance; MACE, major adverse cardiac events; NSTEMI, non–ST-segment–elevation myocardial infarction; STEMI, ST-segment–elevation myocardial infarction.

SUPPLEMENTAL MATERIAL V – Endpoint Prediction Additional Results

Table SV.1 – LDA models coefficients

MODEL	VARIABLES	LDA COEFFICIENTS	CONSTANT
LVEF	LVEF	-0.65	-2.77
CMR	ESV, EDV	1.91, -1.51	-2.93
CMR + Vt	ESV, EDV, RR, $t_{diastolic}$	1.90, -1.48, -0.73, 0.69	-3.00
CMR + Vt_{AI}	ESV, EDV, $Vt_{AI}2$, $Vt_{AI}3$, $Vt_{AI}5$	1.79, -1.42, 0.36, 0.28, 0.37	-3.07
CMR + Strain	ESV, EDV, GLS	1.31, -1.14, 0.52	-3.00
CMR + LV_{3D}	ESV, EDV, C5, C16	1.76, -1.34, -0.33, -0.47	-3.07
ALL	ESV, EDV, Age, Killip	1.55, -1.14, 0.50, 0.53	-3.16
ALL + Vt	ESV, Age, Killip, BSA, RR, $t_{diastolic}$	0.65, 0.58, 0.53, -0.32, -0.91, 0.65	-3.15
ALL + Vt_{AI}	ESV, EDV, Age, Killip, $Vt_{AI}3$, $Vt_{AI}5$	1.52, -1.17, 0.55, 0.51, 0.36, 0.35	-3.26
ALL + Strain	ESV, Age, Killip, BSA, Weight, Vessels, GLS	0.41, 0.44, 0.43, -1.19, 0.83, 0.21, 0.63	-3.24
ALL + LV_{3D}	ESV, EDV, Age, Killip, C5, C16	1.40, -0.98, 0.48, -0.34, -0.43, 0.51	-3.28
All + Vt_{AI} + Strain + LV_{3D}	ESV, EDV, Age, C16, GLS, $Vt_{AI}3$, $Vt_{AI}5$	1.09, -0.88, 0.58, -0.47, 0.58, 0.37, 0.42	-3.35

Coefficients and constant of the resulting LDA models following the backward stepwise variable selection, to assess the additional prognostic contribution of LV contraction unravelling via conventional (Vt) and AI-derived (Vt_{AI}) volume transient; CMR-FT strains (strains); and LV 3D detail patterns (LV_{3D}) metrics on top of considering only CMR biomarkers (CMR) or all the cardiovascular risk factors and patient characteristics of the study (ALL). All the variables are normalized to zero mean and unit variance prior to LDA fitting. The performance of these models is reported in the main manuscript (Table 3).

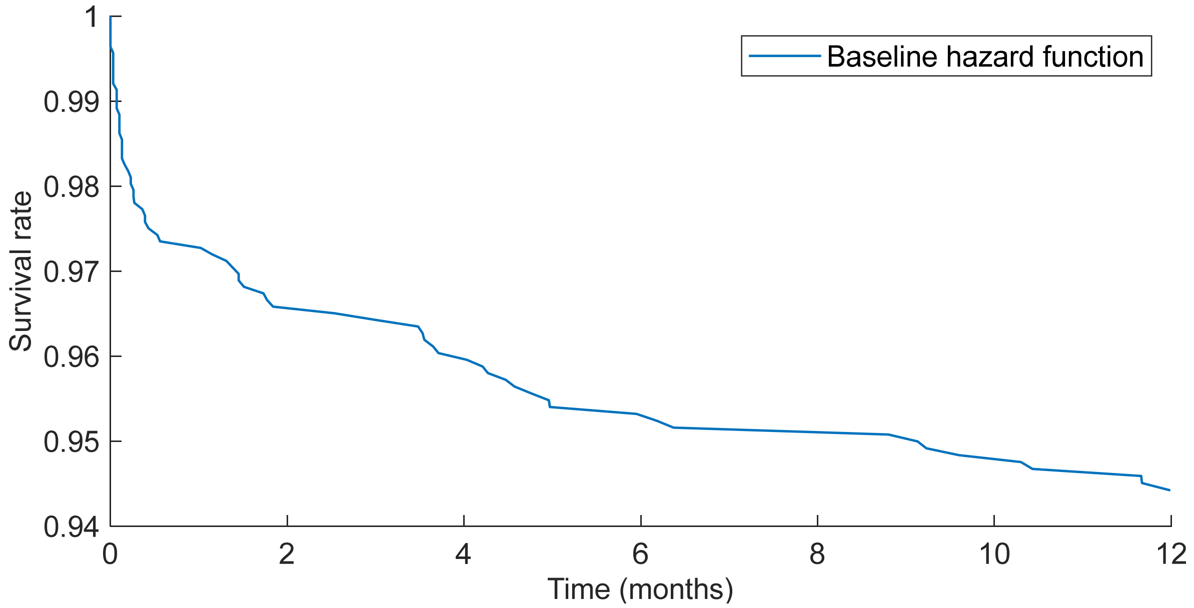

Fig. SV.1 – Underlying baseline hazard function

Table SV.2 – Cox models hazard ratios

MODEL	VARIABLES	HR	P-value
LVEF	LVEF	0.80 (0.74 - 0.87)	< 0.001
CMR	ESV	4.45 (3.01 - 6.58)	< 0.001
	EDV	0.28 (0.18 - 0.43)	< 0.001
CMR + Vt	ESV	4.45 (3.01 - 6.58)	< 0.001
	EDV	0.28 (0.18 - 0.43)	< 0.001
CMR + Vt_{AI}	ESV	4.51 (2.95 - 6.89)	< 0.001
	EDV	0.26 (0.16 - 0.42)	< 0.001
	$Vt_{AI}3$	1.28 (1.05 - 1.55)	0.013
	$Vt_{AI}5$	1.28 (1.05 - 1.56)	0.015
CMR + Strain	ESV	2.79 (1.73 - 4.52)	< 0.001
	EDV	0.38 (0.23 - 0.63)	< 0.001
	GLS	1.69 (1.28 - 2.23)	< 0.001
CMR + LV_{3D}	ESV	4.23 (2.72 - 6.60)	< 0.001
	EDV	0.29 (0.18 - 0.48)	< 0.001
	C16	0.64 (0.51 - 0.80)	< 0.001
	C5	0.76 (0.61 - 0.95)	0.018
ALL	ESV	3.22 (2.12 - 4.91)	< 0.001
	EDV	0.39 (0.24 - 0.63)	< 0.001
	Age	1.55 (1.19 - 2.01)	0.001
	Killip	1.26 (1.10 - 1.45)	0.001
ALL + Vt	ESV	1.56 (1.29 - 1.89)	< 0.001
	Age	1.66 (1.27 - 2.17)	< 0.001
	RR	0.58 (0.44 - 0.78)	< 0.001
	$V_{diastolic\_avg}$	0.09 (0.02 - 0.43)	0.003
	Killip	1.22 (1.05 - 1.41)	0.008
ALL + Vt_{AI}	ESV	3.32 (2.12 - 5.19)	< 0.001
	EDV	0.36 (0.21 - 0.60)	< 0.001
	Age	1.59 (1.21 - 2.07)	< 0.001
	Killip	1.25 (1.08 - 1.43)	0.002
	$Vt_{AI}3$	1.29 (1.06 - 1.57)	0.011
	$Vt_{AI}5$	1.26 (1.03 - 1.54)	0.025
ALL + Strain	GLS	1.78 (1.38 - 2.30)	< 0.001
	Age	1.58 (1.21 - 2.05)	< 0.001
	Killip	1.22 (1.04 - 1.43)	0.013
	BSA	0.72 (0.55 - 0.93)	0.014
	ESV	1.31 (1.06 - 1.63)	0.014
ALL + LV_{3D}	ESV	3.41 (2.15 - 5.40)	< 0.001
	C16	0.65 (0.51 - 0.81)	< 0.001
	EDV	0.39 (0.23 - 0.65)	< 0.001
	Age	1.55 (1.19 - 2.02)	0.001
	C5	0.76 (0.60 - 0.95)	0.019
ALL + Vt_{AI} + Strain + LV_{3D}	GLS	1.78 (1.38 - 2.30)	< 0.001
	C16	0.61 (0.48 - 0.78)	< 0.001
	ESV	2.36 (1.42 - 3.92)	< 0.001
	Age	1.49 (1.15 - 1.95)	0.003
	EDV	0.47 (0.27 - 0.81)	0.007
	$Vt_{AI}5$	1.25 (1.05 - 1.49)	0.012

Hazard ratios, HR (95% confidence interval), and predictor significance, *P*-value, of the resulting Cox multivariate models following the backward stepwise variable selection, to assess the additional prognostic contribution of LV contraction unravelling via conventional (Vt) and AI-derived (Vt_{AI}) volume transient; CMR-FT strains (strains); and LV 3D detail patterns (LV_{3D}) metrics on top of considering only CMR biomarkers (CMR) or the cardiovascular risk factors and patient characteristics of the study (ALL). The performance of these models is summarized in the manuscript (Table 3).

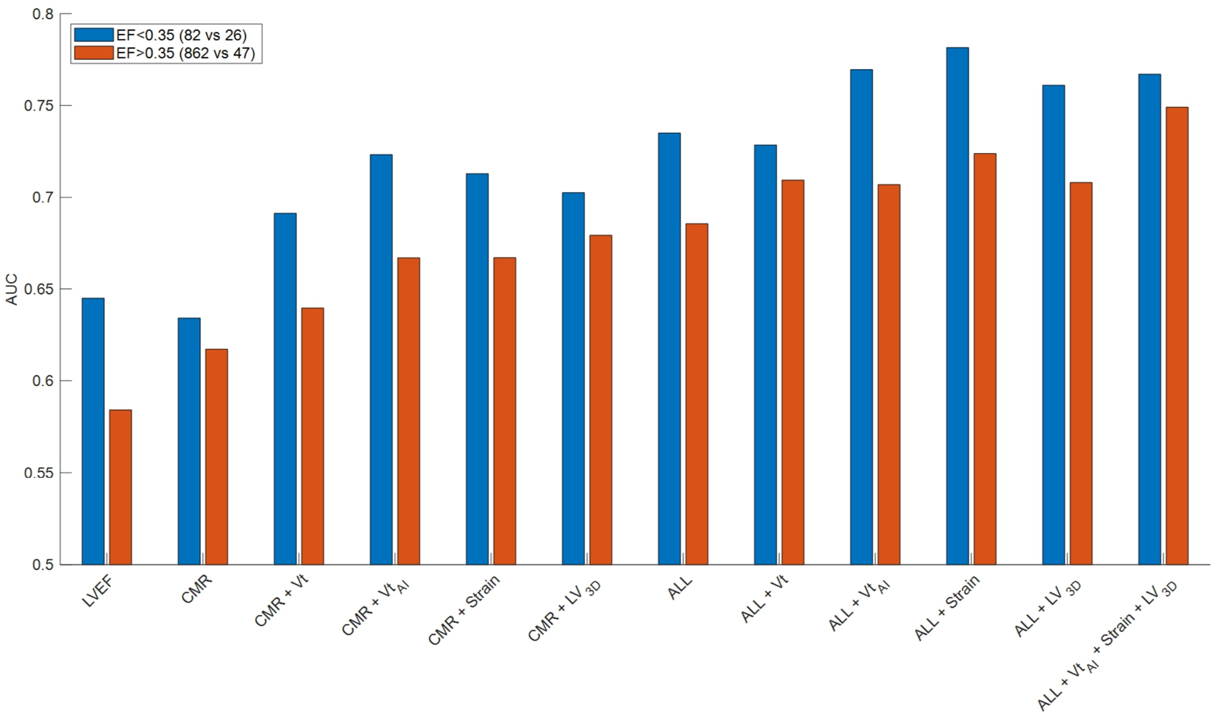

Fig. SV.2 – AUC endpoints prediction results stratifying by LVEF (threshold: 0.35) and applying the LDA models resulting from the backward stepwise analysis (See Table 3 and Table SV.1). The labels are presented as subgroup (MACE vs No MACE cases within the subgroup).

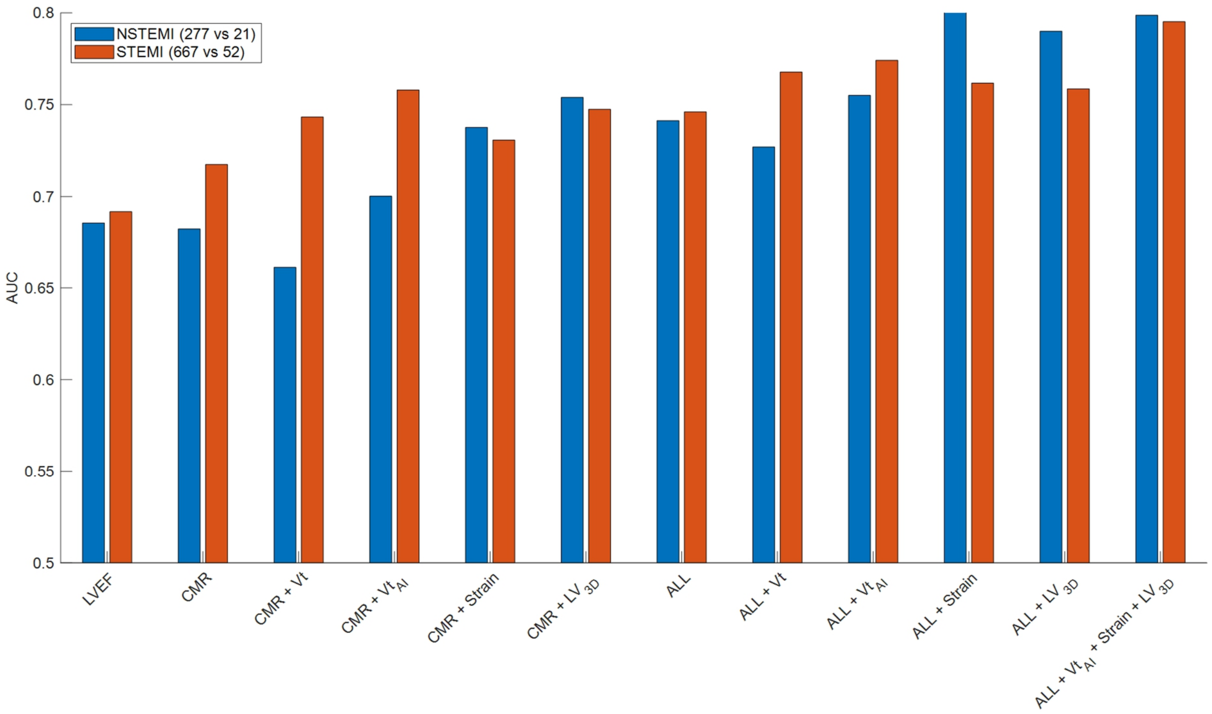

Fig. SV.3 – AUC endpoints prediction results stratifying by infarct aetiology (STEMI vs NSTEMI) and applying the LDA models resulting from the backward stepwise analysis (See Table 3 and Table SV.1). The labels are presented as subgroup (MACE vs No MACE cases within the subgroup).

SUPPLEMENTAL MATERIAL VI – Modes Correlation Additional Results

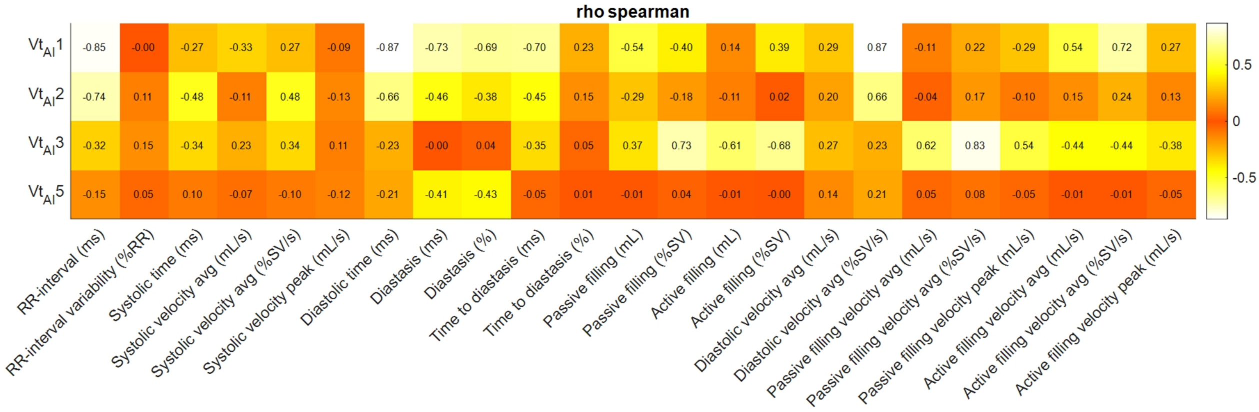

Fig. SVI.1 – Heat map of Spearman correlation coefficients between AI-derived (columns) and conventional (rows) volume transient metrics.

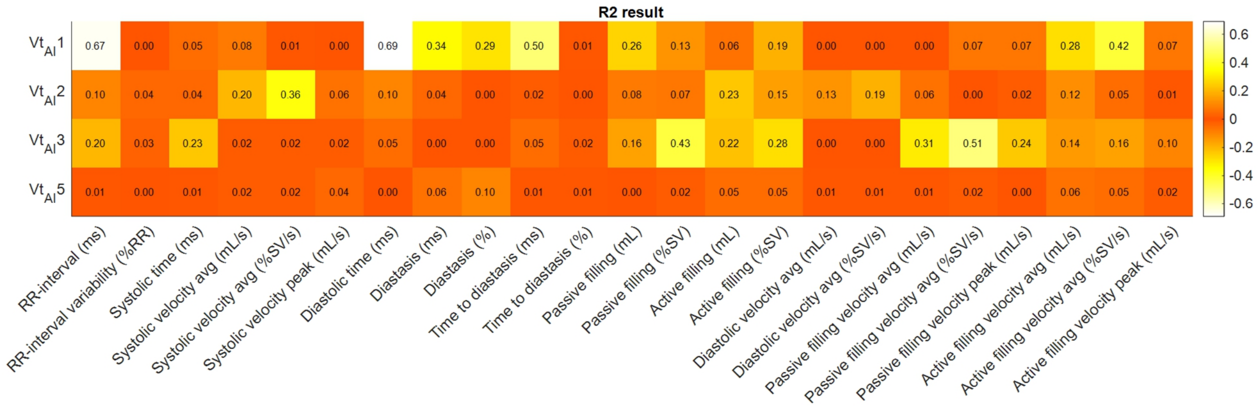

Fig. SVI.2 – Heat map of R2 correlation coefficients between AI-derived (columns) and conventional (rows) volume transient metrics.

SUPPLEMENTAL MATERIAL VII – Systolic vs Diastolic Components

The LV volume temporal transients are split into their systolic and diastolic components using the systolic time as reference. The analysis is repeated for each of the two components independently as described in Methods and Supplemental Material III.

The 95% of the population variance was explained by the first 4 systolic modes of variation (Vt-S_{AI}) and the first 5 diastolic modes of variation (Vt-D_{AI}), respectively. Among them, the LDA stepwise analysis determined modes 2, 4 and 5 (Vt-D_{AI}2, Vt-D_{AI}4 and Vt-D_{AI}5) as relevant to MACE in the diastolic component analysis; and only mode 2 (Vt-S_{AI}2,) in the systolic component scenario (see Fig. SVII.1).

The experiments summarized in Table SVII.1 show that any of the two components significantly contributes to the baseline model (cardiovascular risk factors, basic patient characteristics and established CMR markers), as the LDA stepwise selection demonstrates. This is particularly interesting in the systolic component case study, where the mode Vt-S_{AI}2 is significantly related to MACE in combination with other variables but not if analyzed individually (p = 0.148, see Fig. SVII.1). Nevertheless, only the diastolic component contributes to a significant improvement in prediction performance. This is in line with the results of the entire transient analysis (Vt_{AI}), presented in the main manuscript (see Table 3), where the two modes that contributed the most to the multivariate models were related to diastolic function, that is, Vt_{AI}3 and Vt_{AI}5. It is also sensible that the inclusion of the entire transient provides more additional prognostic value than any of the two components individually analyzed.

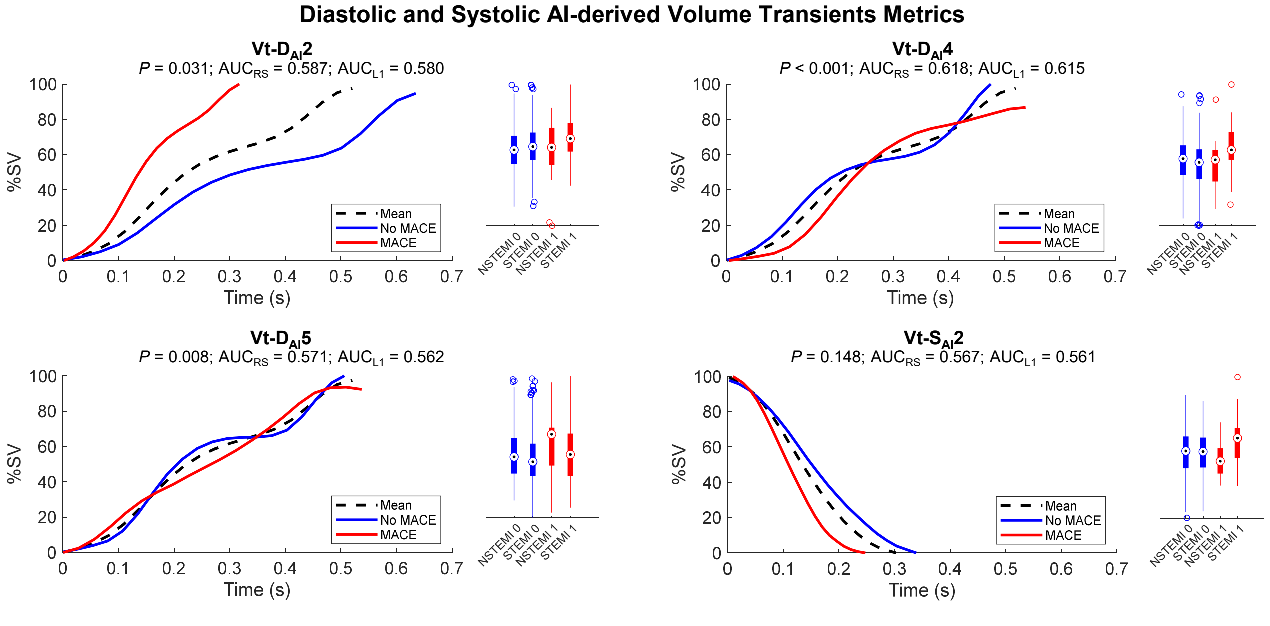

Fig. SVII.1 –AI-derive volume transient features relevant to MACE occurrence prediction, resulting from diastolic component unsupervised learning (Vt-D_{AI}2, Vt-D_{AI} 4, and Vt-D_{AI} 5) and systolic component analysis (Vt-S_{AI}5). The MACE (red, class 1) and No MACE (blue, class 0) representations correspond to the 10th and 90th percentiles in the LDA direction. This allows to visualize the particular pattern or change encode by each of the unsupervised variables (RR-interval, diastasis, etc.) as well as to describe how a representative MACE and No-MACE volume transient components would theoretically look like according to each of these four unsupervised variables. The *P* value, re-substitution and leave-one-out AUCs are presented along each mode as MACE and No-MACE distributions, further stratified into infarct aetiology (STEMI and NSTEMI).

Table SVII.1 – Additional prognostic contribution of AI volume transient components

MODEL	LINEAR SELECTION	AUC_k	AUC_{RS}
ALL	ESV, EDV, Age, Killip	0.729 (0.727 - 0.733)	0.745
ALL + Vt_{AI}	ESV, EDV, Age, Killip, $Vt_{AI}3$, $Vt_{AI}5$	0.746 (0.743 - 0.749)	0.769
ALL + $Vt\text{-}D_{AI}$	ESV, EDV, Age, Killip, $Vt\text{-}D_{AI}4$	0.736 (0.733 - 0.738)	0.751
ALL + $Vt\text{-}S_{AI}$	ESV, EDV, Age, Killip, $Vt\text{-}S_{AI}2$	0.726 (0.723 - 0.730)	0.743

Backward stepwise LDA results of the additional prognostic contribution of the AI-derived volume transient metrics, considering the entire transient (Vt_{AI}), the diastolic component ($Vt\text{-}D_{AI}$) or the systolic component ($Vt\text{-}S_{AI}$), on top of all the cardiovascular risk factors and patient characteristics of the study (ALL). The resulting significant selection of variables is reported along with the predictive performance, expressed as AUC re-substitution (RS) and 10-fold cross-validated (K), computed for a 100 random data splits and presented as median (interquartile range). Killip indicates Killip class on admission.

SUPPLEMENTAL MATERIAL VIII – TRIPOD and STROBE Checklists

Table SVIII.1 – TRIPOD Checklist

DESCRIPTION	ITEM	CHECKLIST	MANUSCRIPT CHECK
**Title & abstract**			
Title	1	Identify the study as developing and/or validating a multivariable prediction model, the target population, and the outcome to be predicted	Title
Abstract	2	Provide a summary of objectives, study design, setting, participants, sample size, predictors, outcome, statistical analysis, results, and conclusions	Abstract
**Introduction**			
Background and objectives	3a	Explain the medical context (including whether diagnostic or prognostic) and rationale for developing or validating the multivariable prediction model, including references to existing models	Introduction (par.1-3)
	3b	Specify the objectives, including whether the study describes the development or validation of the model or both	Introduction (par. 4)
**Methods**			
Source of data	4a	Describe the study design or source of data (e.g., randomized trial, cohort, or registry data), separately for the development and validation data sets, if applicable	Methods (Study Population)
	4b	Specify the key study dates, including start of accrual; end of accrual; and, if applicable, end of follow-up	Methods (Study Population & Study Endpoints)
Participants	5a	Specify key elements of the study setting (e.g., primary care, secondary care, general population) including number and location of centers	Methods (Study Population & CMR Imaging Protocol)
	5b	Describe eligibility criteria for participants	
	5c	Give details of treatments received, if relevant	
Outcome	6a	Clearly define the outcome that is predicted by the prediction model, including how and when assessed	Methods (Study Endpoints)
	6b	Report any actions to blind assessment of the outcome to be predicted	
Predictors	7a	Clearly define all predictors used in developing the multivariable prediction model, including how and when they were measured	Methods (Prognostic Value Assessment & Statistical Analysis), Tables 1 and 2
	7b	Report any actions to blind assessment of predictors for the outcome and other predictors	
Sample size	8	Explain how the study size was arrived at	*
Missing data	9	Describe how missing data were handled (e.g., complete-case analysis, single imputation, multiple imputation) with details of any imputation method	Fig.1 and Results (Patients)
Statistical analysis methods	10a	Describe how predictors were handled in the analyses	Methods (Prognostic Value Assessment & Statistical Analysis)
	10b	Specify type of model, all model-building procedures (including any predictor selection), and method for internal validation	
	10d	Specify all measures used to assess model performance and, if relevant, to compare multiple models	
Risk groups	11	Provide details on how risk groups were created, if done	Methods (Study Endpoints)
**Results**			
Participants	13a	Describe the flow of participants through the study, including the number of participants with and without the outcome and, if applicable, a summary of the follow-up time. A diagram may be helpful	Table 2, Table SIV.1, Fig.1 and Results (Patients)
	13b	Describe the characteristics of the participants (basic demographics, clinical features, available predictors), including the number of participants with missing data for predictors and outcome	
Model development	14a	Specify the number of participants and outcome events in each analysis	Table 2, Fig.1 and Results (Patients)
	14b	If done, report the unadjusted association between each candidate predictor and outcome	
Model specification	15a	Present the full prediction model to allow predictions for individuals (i.e., all regression coefficients, and model intercept or baseline survival at a given time point)	Results and Supplemental Materia V
	15b	Explain how to use the prediction model	
Performance	16	Report performance measures (with CIs) for the prediction model	Table3, Results, and Supplementary Data V

Continued

**Discussion**			
Limitations	18	Discuss any limitations of the study (such as nonrepresentative sample, few events per predictor, missing data)	Discussion (Limitations)
Interpretation	19b	Give an overall interpretation of the results, considering objectives, limitations, results from similar studies, and other relevant evidence	Discussion
Implications	20	Discuss the potential clinical use of the model and implications for future research	Discussion (Impact and Clinical Translation)
**Other information**			
Supplementary information	21	Provide information about the availability of supplementary resources, such as study protocol, Web calculator, and data sets	Supplemental Material
Funding	22	Give the source of funding and the role of the funders for the present study	Funding

Transparent Reporting of a multivariable prediction model for Individual Prognosis Or Diagnosis (TRIPOD) checklist, adapted from (36). Only model development items are included. (*) The size of the study is usually determined based on the variables of interest and their standard deviation. The proposed study cannot benefit from this criterion since the variables (modes of variation) were not defined *a priori*. However, aiming at a margin of error of ≤0.05 in the overall outcome proportion estimate, a mean absolute prediction error of 0.05, a desired shrinkage ≤10% and conservative anticipated Cox-Snell R squared statistic of 0.2, and given the approximate MACE incidence of 7% and the number of predictors indicated in the manuscript, the actual size of the study is superior to the trial target size retrospectively determined by any of the 4 proposed calculation methods explained in Riley et al. (26). Par. indicates paragraph.

Table SVIII.2 – STROBE Checklist

DESCRIPTION	ITEM #	RECOMMENDATION	PAGE #
**Title and abstract**			
Title & abstract	1	(*a*) Indicate the study's design with a commonly used term in the title or the abstract	1, 3
		(*b*) Provide in the abstract an informative and balanced summary of what was done and what was found	3
**Introduction**			
Background/ rationale	2	Explain the scientific background and rationale for the investigation being reported	5
Objectives	3	State specific objectives, including any prespecified hypotheses	3, 6
**Methods**			
Study design	4	Present key elements of study design early in the paper	6 & Fig. 1
Setting	5	Describe the setting, locations, and relevant dates, including periods of recruitment, exposure, follow-up, and data collection	7, 8
Participants	6	(*a*) Give the eligibility criteria, and the sources and methods of case ascertainment and control selection. Give the rationale for the choice of cases and controls	7
		(*b*) For matched studies, give matching criteria and the number of controls per case	-
Variables	7	Clearly define all outcomes, exposures, predictors, potential confounders, and effect modifiers. Give diagnostic criteria, if applicable	7, 10 & Table 1
Data sources/ measurement	8	For each variable of interest, give sources of data and details of methods of assessment (measurement). Describe comparability of assessment methods if there is more than one group	7-11
Bias	9	Describe any efforts to address potential sources of bias	10, 11
Study size	10	Explain how the study size was arrived at	*
Quantitative variables	11	Explain how quantitative variables were handled in the analyses. If applicable, describe which groupings were chosen and why	10, 11
Statistical methods	12	(*a*) Describe all statistical methods, including those used to control for confounding	10, 11
		(*b*) Describe any methods used to examine subgroups and interactions	11
		(*c*) Explain how missing data were addressed	11
		(*d*) If applicable, explain how matching of cases and controls was addressed	-
		(*e*) Describe any sensitivity analyses	10, 11
**Results**			
Participants	13	(a) Report numbers of individuals at each stage of study—eg numbers potentially eligible, examined for eligibility, confirmed eligible, included in the study, completing follow-up, and analysed	12 & Fig. 2
		(b) Give reasons for non-participation at each stage	12
		(c) Consider use of a flow diagram	Fig. 2
Descriptive data	14	(a) Give characteristics of study participants (eg demographic, clinical, social) and information on exposures and potential confounders	Table 2, Table SIV.1
		(b) Indicate number of participants with missing data for each variable of interest	Table 2, Table SIV.1
Outcome data	15	Report numbers in each exposure category, or summary measures of exposure	Table 2, Table SIV.1

Continued

Main results	16	(*a*) Give unadjusted estimates and, if applicable, confounder-adjusted estimates and their precision (eg, 95% confidence interval). Make clear which confounders were adjusted for and why they were included	Table 2, Table 3, Table SIV.1
		(*b*) Report category boundaries when continuous variables were categorized	Table 2, Table 3, Table SIV.1
		(*c*) If relevant, consider translating estimates of relative risk into absolute risk for a meaningful time period	Table 2, Table 3, Table SIV.1, Fig. 5
Other analyses	17	Report other analyses done—eg analyses of subgroups and interactions, and sensitivity analyses	Fig. 4 & Supplemental Material
**Discussion**			
Key results	18	Summarise key results with reference to study objectives	15, 21
Limitations	19	Discuss limitations of the study, taking into account sources of potential bias or imprecision. Discuss both direction and magnitude of any potential bias	20
Interpretation	20	Give a cautious overall interpretation of results considering objectives, limitations, multiplicity of analyses, results from similar studies, and other relevant evidence	17-20
Generalisation	21	Discuss the generalisability (external validity) of the study results	15-17
**Other information**			
Funding	22	Give the source of funding and the role of the funders for the present study and, if applicable, for the original study on which the present article is based	22

Strengthening the Reporting of Observational Studies in Epidemiology (STROBE) checklist for case-control studies, adapted from (40). (*) The size of the study is discussed in Table SVIII.1.
